# Oxygen Consumption in Filamentous Pellets of *Aspergillus niger*: Microelectrode Measurements and Modeling

**DOI:** 10.1002/bit.28874

**Published:** 2024-11-04

**Authors:** Charlotte Deffur, Anna Dinius, Julian Pagel, Henri Müller, Stefan Schmideder, Heiko Briesen, Rainer Krull

**Affiliations:** ^1^ School of Life Sciences Weihenstephan, Chair of Process Systems Engineering Technical University of Munich Freising Germany; ^2^ Institute of Biochemical Engineering Technische Universität Braunschweig Braunschweig Germany; ^3^ Center of Pharmaceutical Engineering Technische Universität Braunschweig Braunschweig Germany

**Keywords:** 3D image analysis, active part percentage, microelectrode technique, morphology, oxygen consumption, parameter estimation, X‐ray microcomputed tomography

## Abstract

Filamentous fungi cultivated as biopellets are well established in biotechnology industries. A distinctive feature of filamentous fungi is that hyphal growth and fungal morphology affect product titers and require tailored process conditions. Within the pellet, mass transfer, substrate consumption, and biomass formation are intricately linked to the local hyphal fraction and pellet size. This study combined oxygen concentration measurements with microelectrode profiling and three‐dimensional X‐ray microtomography measurements of the same fungal pellets for the first time. This allowed for the precise correlation of micromorphological information with local oxygen concentrations of two *Aspergillus niger* strains (hyperbranching and regular branching). The generated results showed that the identified oxygen‐penetrated outer pellet regions exhibited a depth of 90–290 µm, strain‐specific, with the active part percentage in the pellet ranging from 18% to 69%, without any difference between strains. Using a 1D continuum diffusion consumption model, the oxygen concentration in the pellets was computed depending on the local hyphal fraction. The best simulation results were achieved by individually estimating the oxygen‐related biomass yield coefficient of the consumption term within each examined pellet, with an average estimated value of 1.95 (± 0.72) kg biomass per kg oxygen. The study lays the foundation for understanding oxygen supply in fungal pellets and optimizing processes and pellet morphologies accordingly.

## Graphical abstract

We isolated pellets from *Aspergillus niger* cultivations and measured local oxygen concentrations using a microelectrode. These identical pellets underwent structural analysis via microcomputed tomography to produce 3D images. By integrating data from both methods, we analyzed hyphal oxygen supply and developed a model to simulate oxygen concentration within a single pellet.

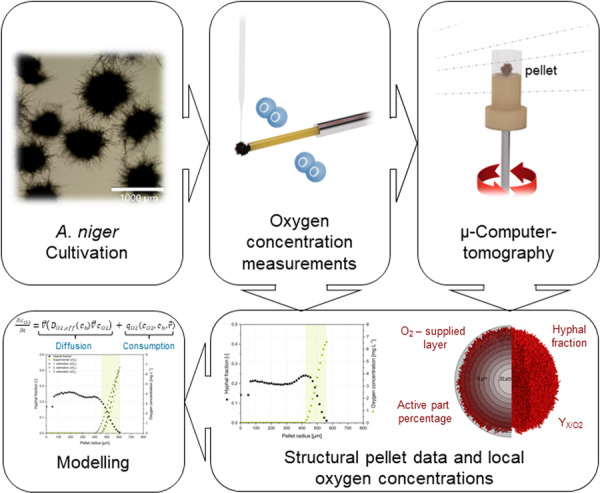

## 1 Introduction

In nature, filamentous fungi excel in decomposition and biosynthesis, which provides a platform to be harnessed for industrial‐scale production of various substances relevant to pharmaceutical, chemical, or food industries (Meyer et al. 2021). Usually, industrial‐scale submerged cultivation of filamentous microorganisms, such as *Aspergillus niger*, exhibits various visible growth structures due to their multicellular character. These structures are collectively referred to as macromorphology and range from loosely dispersed mycelium to dense, nearly spherical pellets. The presence of dispersed mycelium in the culture broth bears several disadvantages on the processing level, including poor mixing due to increased viscosity (Dinius, Kozanecka et al. 2024). Therefore, pellet morphology is often preferred over dispersed mycelium to overcome mixing challenges during the cultivation process. However, in the case of pellet morphology, dense hyphal structures can impede the inner mass transport of substrates, resulting in limitations in the pellet center. This can be unfavorable for the synthesis of certain product types (Hille et al. 2005; Krull et al. 2013). Controlling the growth of pellets is challenging and is the subject of current research. Several factors have already been identified to affect this process, e.g., spore concentration, spore agglomeration, pH value, oxygen availability, shear stress induced by fluid movement, air bubbles, and the stirrer (Böl et al. 2021; Walisko et al. 2015). At the micromorphological level, pellet growth is characterized by the apical hyphal growth and branching of the hyphae, which especially depends on the available substrate concentration within the pellet. The availability of substrates in the pellet, in turn, depends on the hyphal density in the pellet, the associated diffusion processes, and the substrate consumption of the cells in the outer pellet region (Hille et al. 2005; Schmideder et al. 2021; Veiter, Rajamanickam, and Herwig 2018). Furthermore, in loosely structured pellets, particularly under turbulent flow conditions, convective transport contributes significantly to substrate supply in addition to diffusive transport (Hille et al. 2009).

Regarding process and production enhancement, some studies employ innovative morphological engineering techniques to adjust morphology development precisely. This includes, for instance, cultivations using microparticles or the alteration of the osmolality to loosen the hyphal structure and control the pellet diameter (Laible et al. 2021; Dinius, Kozanecka et al. 2024). For an effective cultivation process or their improvement via morphology engineering, a detailed characterization of pellets on the micro‐ and macromorphological level is obligatory. A common method involves capturing microscopic images of samples to obtain information about the macromorphological state of the population during cultivation. Using these 2D images, quantitative size and shape descriptors (Euclidean shape descriptors) such as the projected area, diameter, aspect ratio, circularity, and solidity of the periphery can be determined. The dimensionless morphology number (MN) summarizes this information, indicating the roundness and smoothness of the pellet (Wucherpfennig, Hestler, and Krull 2011). To assess information about the hyphal density within the pellet, pellet slicing and subsequent confocal laser scanning microscopy (CLSM) can be conducted (Driouch, Sommer, and Wittmann 2010; Hille et al. 2005; Lin, Scholz, and Krull 2010). Furthermore, by including live/dead staining steps followed by CLSM, information about the viability of cells within the pellet can be obtained. In these images, dead biomass can be distinguished from living biomass using different stains (Dinius, Kozanecka et al. 2024; Schrinner et al. 2020). It has been revealed that oxygen is the predominant limiting nutrient in fungal pellets (Hille et al. 2005 and 2009; Veiter, Rajamanickam, and Herwig 2018). Therefore, the pioneering works by Cronenberg et al. (1994) and Wittier et al. (1986) inserted microelectrodes into *Penicillium chrysogenum* pellets to determine local oxygen concentrations. Hille et al. (2005) also conducted oxygen concentration measurements using microelectrodes, this time in *A. niger* pellets. In that study, attempts were made to correlate oxygen concentration profiles with hyphal densities of the pellets. Thereby, the hyphal density was determined two‐dimensionally via CLSM images, resulting in inaccurate and significantly elevated measurements. However, these experimental methods cannot provide holistic information on individual spherical pellets and, at the same time, achieve a significant analytical throughput.

In addition to experimental studies, there is a focus on the computation of growth, the hyphal structure, and the oxygen supply within the pellet to generate a comprehensive process understanding and establish a basis for optimizations (Böl et al. 2021). In an early study, the cubic root law was defined, describing the growth of pellets as an exponential biomass increase within an active outer spherical shell. In this estimation, the density in the pellet is assumed to be constant (Emerson 1950; Marshall and Alexander 1960; Pirt 1966). Cui et al. (1998) developed a mathematical model of growth and deactivation kinetics, incorporating factors such as oxygen transfer, stirring intensity, dissolved oxygen tension, pellet size, mycelial formation, and substrate and oxygen consumption. However, in that model, the hyphal density within the pellet was estimated to be spatially constant, and due to a lack of experimental data, it was considered a linear function of time. Rinas et al. (2005) developed a one‐dimensional pellet model to investigate the effects of pellet size and the number of pellets in cultivation on glucose oxidase production. Although the model provides a detailed description of consumption kinetics, it lacks a comprehensive representation of effective oxygen diffusion within the pellet. The model treats hyphal density and effective oxygen diffusion as constants. This assumption does not apply to real pellets, leading to intrinsic inaccuracies in the results. A few studies have considered radially resolved local properties such as hyphal length, tips, biomass, and oxygen concentrations. Buschulte's continuum approach (Buschulte 1992) already determined the temporal changes in hyphal length, tips, and oxygen concentrations through coupled partial differential equations, which was simplified by Meyerhoff, Tiller, and Bellgardt (1995) into a shell model due to significant computational load, where spherical shells assumed constant values for hyphal length and tip concentrations. Stochastic models by Yang et al. (1992) and Celler et al. (2012) describe biomass growth through a sequence of random formations of new hyphal elements (apical growth and branching) in 3D space, thus generating pellet structures with micromorphological information. However, at the time of their development, these models lacked sufficient validation procedures, leading to a stagnation in their advancement.

In summary, it can be observed that a detailed characterization of pellet morphology is primarily lacking in the description of consumption and production processes in filamentous fungal pellets. Therefore, in this study, a novel microcomputed tomography (µCT) measurement (Müller et al. 2023; Schmideder et al. 2021; Schmideder, Barthel, Friedrich et al. 2019; Schmideder, Barthel, Müller et al. 2019) was combined with the oxygen microelectrode technique (Hille et al. 2005) to gain new insights and address the knowledge gap. This recently introduced µCT method used for fungal pellets can be utilized for recording 3D images. Its advantages lie in noninvasiveness and the ability to measure the pellet structure as a whole. Using these µCT data, it can be shown that the hyphal density within the pellet is not constant (Müller et al. 2023; Schmideder, Barthel, Friedrich et al. 2019). Further, a study was published in which the effective diffusion through the mycelial network of various filamentous microorganisms was described as a function of the local hyphal fraction (Schmideder et al. 2021).

In the current study, the *A. niger* SKAn1015 strain (Driouch, Sommer, and Wittmann 2010; Zuccaro et al. 2008) and hyperbranching *A. niger* MF22.4 strain (Fiedler et al. 2018) were used for exemplification. Based on µCT measurements, the local and global morphological characteristics inside individual pellets were investigated. Before analyzing the morphology, the oxygen profile was measured in the same pellets. With these data, critical reaction kinetic parameters for the oxygen consumption rate and further information about the intercorrelation of pellet structure and oxygen supply were estimated. It is important to emphasize that our primary focus is not to compare the two strains directly but to use them as separate examples to validate our combined method.

## 2 Materials and Methods

### 2.1 Cultivation and Analytical Measurements

In this study, the recombinant fructofuranosidase‐producing strain *A. niger* SKAn1015 (Driouch, Sommer, and Wittmann 2010; Zuccaro et al. 2008), in the following referred to as strain R (regular branching), and the glucoamylase‐producing *A. niger* MF22.4 (Fiedler et al. 2018), in the following referred to as strain H (hyperbranching), were utilized. A detailed protocol for spore inoculum preparation can be found in Dinius, Müller et al. (2024). Spore inoculum was prepared by agar plate cultivation and subsequent harvesting with 0.9% NaCl solution. The inoculum was then used for cultivations in 250 mL Erlenmeyer flasks equipped with three defined baffles. The inoculation concentration was set to 1 × 10^6^ spores mL^−1^ for both strains. *A. niger* SKAn1015 was cultivated in 50 mL of sterile defined minimal medium according to Driouch, Sommer and Wittmann (2010), while *A. niger* MF22.4 grew on a semi‐complete medium, according to Fiedler et al. (2018). Incubation was carried out on a rotary shaker (Certomat BS‐1, Sartorius, Göttingen, Germany) in the dark at 37°C, 120 min^−1^ with a 50 mm amplitude until the stationary growth phase was reached. Samples were isolated by terminating the cultivation of the entire shake flask at ongoing time points (**R1**: 28, 30, 32, 40, 42, 45, 47 h; **R2**: 28, 29, 32, 34, 36, 42, 44, 46, 48 h; **H**: 17, 19, 22, 25, 29 h) during cultivations to cover the entire growth curve. Since previous test cultivations of H showed a high degree of consistency in terms of cultivation and sample homogeneity, only one run was used for full analysis.

For each sample, the following analytical steps were performed. The quantification of cell dry weight using filter papers and the glucose analysis via HPLC was conducted as described by Schrinner et al. (2020). Pellet size analysis was carried out microscopically, following the procedure outlined in Dinius et al. (2023), and is depicted as the area‐equivalent spherical diameter (AESD). The MN was calculated using Equation (1), where the parameters correspond to microscopy image data analyzed with ImageJ 1.54d software (National Institutes of Health, Bethesda, USA) in 8‐bit format (Wucherpfennig, Lakowitz, and Krull 2013).

(1) $$\mathrm{MN}=\frac{2∙\sqrt{\mathrm{Area}}∙\mathrm{Solidity}}{\sqrt{\pi }∙{\mathrm{Feret}}^{\prime }s\;Diameter∙Aspect\;Ratio}.$$

### 2.2 Viability Staining and Pellet Slicing

Viability staining of pellets was conducted according to Schrinner et al. (2020). In brief, individual pellets were isolated from the culture broth, washed with deionized water, and transferred to the staining solution containing 1.5 µL SYTO9 (3.34 mM, DMSO) and Propidium iodide (PI, 20 mM, DMSO) (both Molecular Probes, Eugene, USA) in 1 mL salt solution (0.31 g L^−1^ ammonium chloride, 4.33 g L^−1^ disodium hydrogen phosphate, 0.13 g L^−1^ potassium chloride, 3.04 g L^−1^ sodium dihydrogen phosphate dihydrate). After incubation (20 min, room temperature in the dark) in an overhead shaker (Intelli‐Mixer RM‐2 M, LTF Labortechnik, Wasserburg, Germany) and a subsequent washing step, the pellets were incubated (20 min) and frozen at −20°C in liquid cryosectioning medium (Neg‐50, Richard Allan Scientific, USA).

Pellet slicing (50 µm thickness) was performed as described in Dinius et al. (2023) using a cryomicrotome (HM550, Microm, Neuss, Germany). Pellet cross‐sections from the equatorial region (slice of largest diameter) were transferred to a microscope slide and examined using CLSM (C2si, Nikon Instruments, Amsterdam, The Netherlands) with appropriate magnification, similar to the procedure described in Schrinner et al. (2020). A deviation of living and dead cell mass can be made by visualizing SYTO9 with a laser set at 510–540 nm and PI at 620–650 nm. Consequently, viable biomass in the pellets appeared green due to the intercalation of SYTO9 into the nucleic acid of all cells. In the pellets, areas containing dead biomass emit a red fluorescence because PI selectively penetrates cells with compromised cell walls. Thus, PI intercalates into the nucleic acid of dead cells and outshines SYTO9 due to its stronger affinity with nucleic acid (Stocks 2004).

### 2.3 Oxygen Profiling and Pellet Preparation

**Figure 1:**
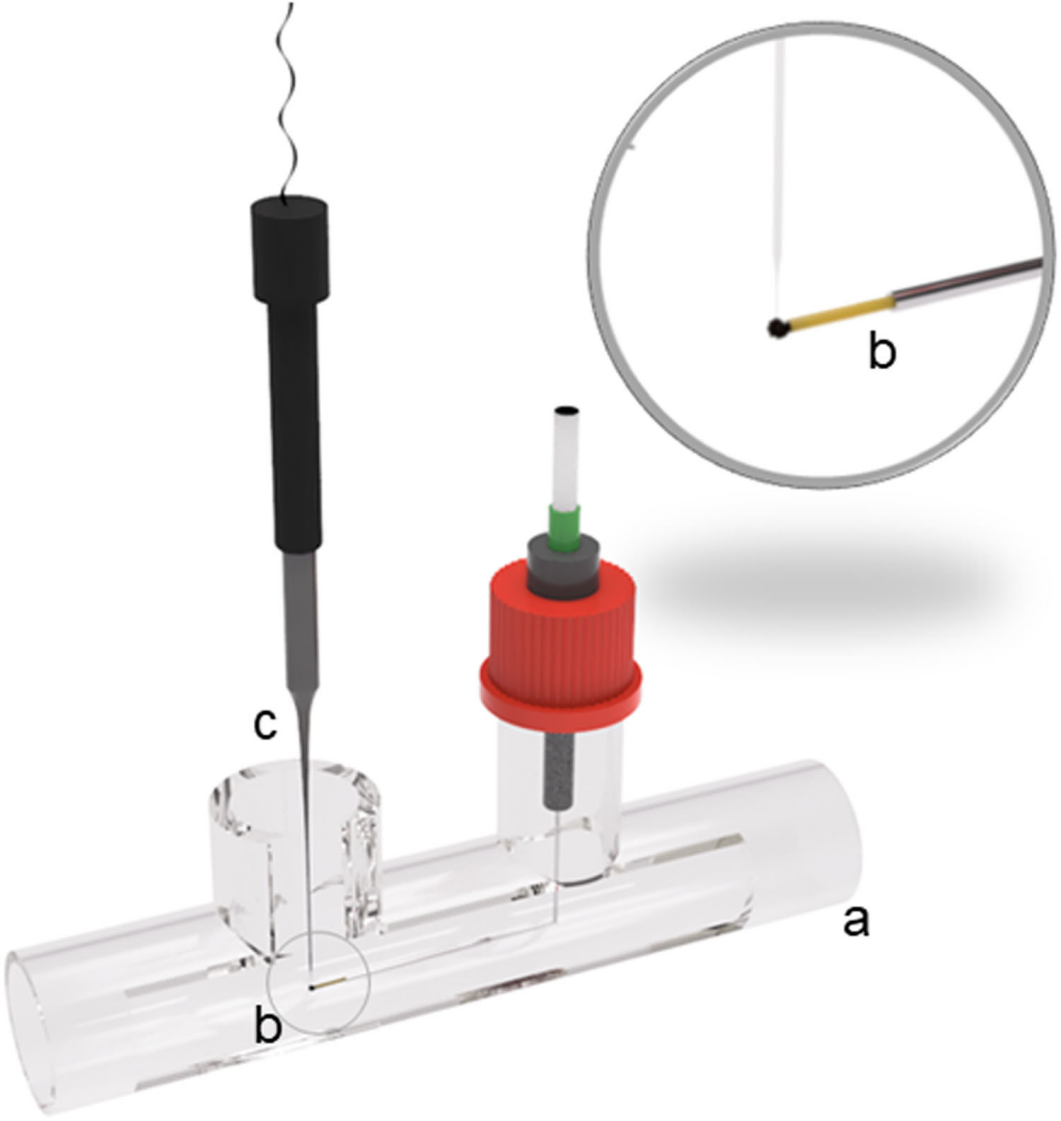
Depiction of the experimental setup for oxygen microprofiling in pellets of filamentous fungi. Pellets are analyzed (a) in a glass tube under laminar flow conditions. Pellet is fixated in the center of the glass tube (b) using a suction cannula (c) The oxygen micro‐sensor (10 µm maximum tip size) is attached to a software‐controlled micromanipulator (MM, not shown). Oxygen concentration is measured in 10 µm step sizes inside the pellet.

For oxygen‐microelectrode measurements, at least three nearly spherical (visually evaluated) pellets with a minimum diameter of 500 µm were isolated for each sample point. Their size was roughly estimated using microscopy (EVOS XL, AMG, Bothell, WA, USA) and image analysis (ImageJ 1.54d, National Institut of Health, Bethesda, USA). Oxygen concentration measurements were conducted using a similar experimental setup and method described by Dinius et al. (2023) (Figure 1).

Briefly, the pellets were initially fixed in the center of a horizontally installed 1.7 m glass tube (*d_i_* = 24 mm), in which constant temperature, adjustable laminar flow conditions, and oxygen saturation were maintained (due to circulating diluted medium). The medium in the glass tube was saturated with compressed air, reaching a saturation of 6.7 mg L^−1^ at 37°C at the start of the experiment. However, since the measurements were conducted in an unsterile environment, the oxygen concentration decreased slightly over time due to contamination. At the latest, when the concentration reached a minimum of 4.8 mg L^−1^, the medium was changed. To ensure a comprehensive coverage of pellet sizes, a suction technique was employed for pellet fixation during profiling. The pellet was drawn in (Δ*p* = 0.19 bar) using a bent cannula (*d_i_* = 0.2 mm, Microcaps 1 µL micropipettes, Drummond Scientific Company, Broomall, USA) with the assistance of a vacuum pump (SG O, Schego, Offenbach, Germany). The pressure was monitored using a digital manometer and controlled with an additional valve. Once the pellet was positioned in the flow tube, the pressure was reduced to 0.07 bar to prevent interference with the natural oxygen supply in the pellet. Initial pressure‐based experiments showed minimal impact on the oxygen profiles of small pellets with a high ratio between the inner cannula and pellet diameter. Suction could have increased the concentration at the pellet center, specifically in the area in front of the cannula. Aside from the slight possibility that oxygen profiles were affected in the pellet center, the suction method was the only viable option for studying relatively small pellets. This method was also less disruptive to the pellet structure and more time‐efficient than the loop‐fixation technique used in previous work (Dinius et al. 2023). The generated data are critically examined for irregularities, such as unrealistically high concentrations in the pellet center.

A Clark‐type oxygen microsensor (Ox‐10) with a maximum tip size of 10 µm was used for profiling to ensure minimal invasive pellet penetration. The microsensor was clamped in a micromanipulator (MM33‐M), which is connected to a motorized micromanipulator stage (MMS) and controlled by a motor controller (MOTCON). For data acquisition, a picoammeter (PA2000), a two‐channel A/D converter (ADC 216), and the control software (SensorTraceSuite v3.3.175) were employed (all items purchased from Unisense A/S, Aarhus, Denmark).

Using a board‐level camera, the microsensor was centrally positioned at the upper border of the pellet. Profiling was conducted by a software‐controlled, stepwise 10 µm insertion (as recommended by the manufacturer) of the microsensor into the equator area of the pellet perpendicularly to the flow direction in the flow cell. After each step, the microsensor rested for 3 s before the local oxygen concentration was measured (averaged over 3 s for each step) until the pellet center was reached (on the basis of the microscopically estimated pellet radius). A minimum of three replicates were conducted for each pellet to derive the mean profile. After profiling, the pellets were washed and freeze‐dried following a previously established protocol (Schmideder, Barthel, Friedrich et al. 2019).

### 2.4 X‐Ray Microcomputed Tomography

**Figure 2:**
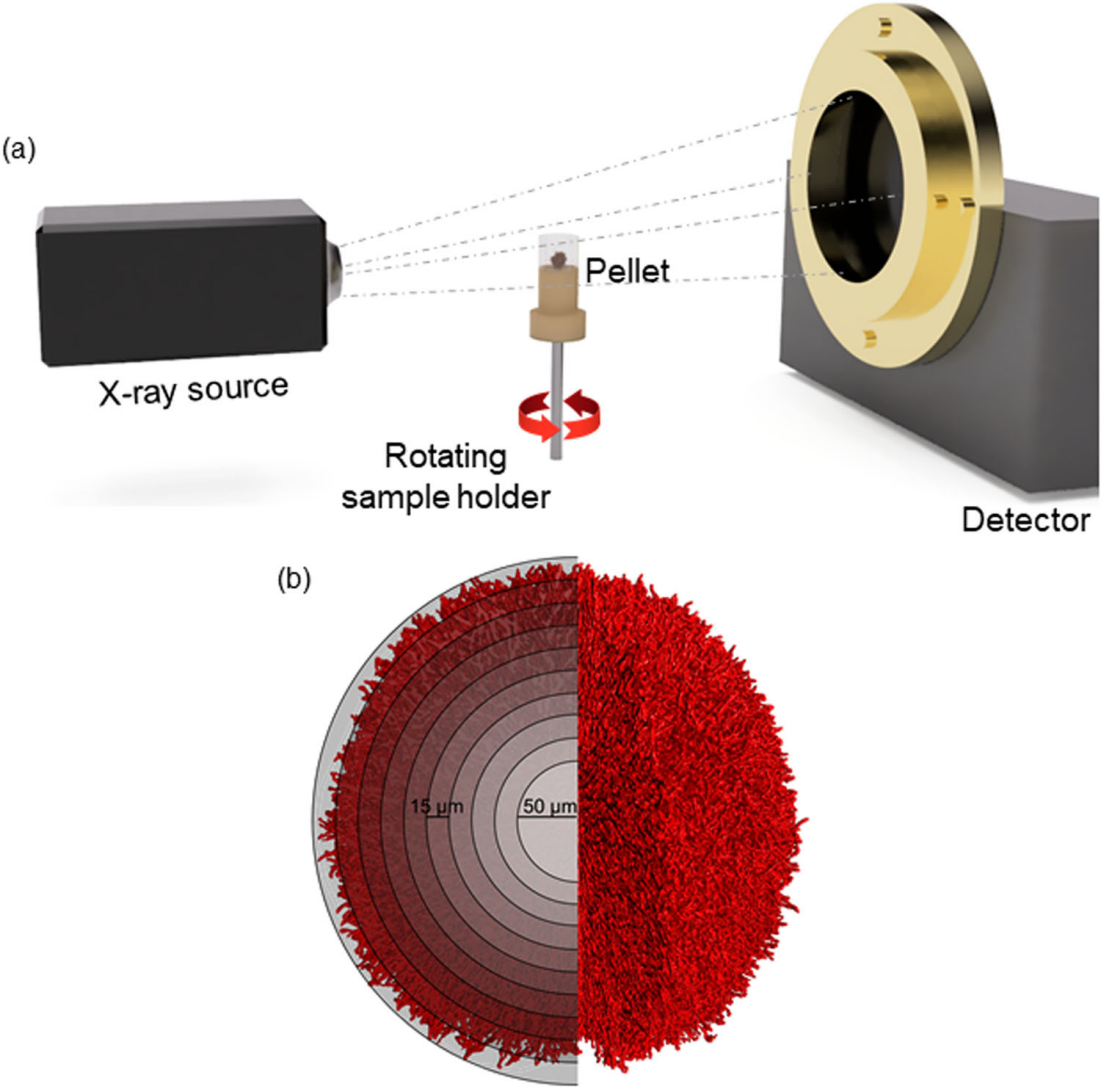
Representation of the X‐ray microtomography measurement. (a) Description of the setup for µCT measurement of filamentous fungal pellets. (b) Schematic representation of the evaluation of micromorphological information within spherical shells (15 µm thickness) around the center of mass.

A custom‐built X‐ray microtomography system (XCT‐1600HR; Matrix Technologies, Feldkirchen, Germany) was used for generating three‐dimensional images of the freeze‐dried fungal pellets based on the earlier described method in Schmideder, Barthel, Friedrich et al. (2019). For an exact assignment to the oxygen data, only one freeze‐dried pellet at a time was carefully placed in the sample holder and measured. A previously developed sample holder (Müller et al. 2023) was utilized. At least 2000 2D projections were obtained from various angles and reconstructed into a 3D image (Figure 2a) using a custom software (Matrix Technology) based on CERA (Siemens). The voxel size of the images was 1 µm.

### 2.5 Image Processing

The images were imported into VGSTUDIO MAX (version 3.2; Volume Graphics GmbH), and the pellet was cut out by manually creating a region of interest (ROI) that contains the pellet but not the sample holder. The selected ROI was extracted, saved as TIFF files, and imported into MATLAB (version R2021a; MathWorks). Processing the 3D images and determination of the hyphal fraction was carried out as in Schmideder et al. (2019). First, gray level images were binarized by calculating a threshold using the Otsu's method. This allowed for distinguishing between hyphal material and air. The local hyphal volume was then determined in shells with a width of 15 µm around a sphere in the mass center of the pellet, which has a radius of 50 µm (Figure 2b). The hyphal fraction was calculated as the ratio of the hyphal volume in the shell to the volume of the total shell. The axis ratio and the convex hull of the binarized image were determined using MATLAB functions (regionprops3, convhull). Pellet sphericity was calculated by dividing the surface area of the volume‐equivalent sphere by the surface area of the convex hull.

### 2.6 Data Processing

The local hyphal fraction was given for defined distances to the mass center of the pellet. Oxygen measurements yielded concentration values at specified depths controlled by the micromanipulator. The determined oxygen concentration had to be converted into values corresponding to defined distances from the pellet center to facilitate data processing. No direct reference to the pellet center was given, so consequently, the pellet border was determined based on the oxygen concentration profile. The border position was defined at the location where 95% of the maximum oxygen concentration was achieved. This approach was supported by the lack of a clear phase boundary between the solid pellet volume and the surrounding liquid culture medium phase due to the loose hyphal structure. Similarly, the pellet border in the µCT data was defined as the cutoff at which the hyphal fraction decreased to 0.005 instead of 0 to reduce the influence of a single hyphae protruding from the pellet. The pellet border as a fixed point in the hyphal fraction and oxygen data enabled the alignment. Maximum gradients in the experimental data were calculated with central second‐order finite differences using MATLAB. The active part percentage (APP) of a single pellet was calculated by determining the proportion of the hyphal volume within the oxygen‐supplied region to the total hyphal volume in the pellet.

### 2.7 Statistical Analyses

Single‐factor analyses of variance (ANOVA) were conducted to verify the results´ statistical reliability. The statistical test (*α* = 0.05) was performed using the MATLAB function anova1. A *p*‐value lower than 0.05 (an *F*‐value higher than the critical *F*‐value, respectively) represents a statistically significant difference between the two groups.

### 2.8 Mathematical Modeling

A continuum‐scale model was used to simulate the oxygen concentration in the pellet as a function of the local hyphal fraction $c_{h}(r)$, which is dependent on the pellet radius r. Using this method, the morphological patterns within the pellet were incorporated into the calculation of oxygen transport and consumption. To simplify the 3D system into a 1D model, a spherically symmetrical hyphal fraction and oxygen concentration were assumed. This eliminates angular dependencies. Equation (2) describes oxygen consumption and diffusion as the only transport mechanism within the pellet (Buschulte 1992; Meyerhoff, Tiller, and Bellgardt 1995).

(2) $$\frac{\partial c_{O_{2}}\left(r\right)}{\partial t}=\frac{1}{r^{2}}\left(\frac{\partial }{\partial r}\left(D_{O_{2},\mathrm{eff}}\left(r\right)r^{2}\frac{\partial c_{O_{2}}\left(r\right)}{\partial r}\right)\right)-q_{O_{2}}\left(r\right).$$

The pellet radius r (spatial direction, in meters m) is equivalent to the distance to the mass center. The effective diffusion coefficient of oxygen $D_{O_{2},\mathrm{eff}}$ ($m^{2}h^{-1}$) was calculated with the equation developed by Schmideder et al. (2021):

(3) $$D_{O_{2},\mathrm{eff}}\left(r\right)=D_{O_{2},\mathrm{bulk}}{\left(1-c_{h}\left(r\right)\right)}^{a},$$

where $D_{O_{2},\mathrm{bulk}}$ ($m^{2}h^{-1}$) is the diffusion coefficient in the bulk, $c_{h}$ the local hyphal fraction (dimensionless) and the exponent a the hindrance factor (dimensionless). Oxygen is considered the only limiting substrate, with glucose present in excess during oxygen measurement. Additionally, oxygen has a relatively low water solubility compared to other substrates, making its presence in excess challenging. However, further substrate dependencies can be integrated into the model (Rinas et al. 2005). The specific oxygen consumption term $q_{O_{2}}$can be described by the oxygen consumption for the formation of new biomass, as in the case of Celler et al. (2012) and Rinas et al. (2005):

(4) $$q_{O_{2}}\left(r\right)=\frac{\rho_{h}}{Y_{X/O_{2}}}\frac{c_{O_{2}}\left(r\right)}{K_{\mathrm{XO}}+c_{O_{2}}\left(r\right)}{µ}_{\max}c_{h}\left(r\right),$$

where $\rho_{h}$ $\left(\mathrm{kg}m^{-3}\right)$ is the hyphal density, ${µ}_{\max}$ ($h^{-1}$) the maximum growth rate, $Y_{X/O_{2}}$ the yield coefficient $(\mathrm{kg}{\mathrm{kg}}^{-1}$) and $K_{\mathrm{XO}}$ ($\mathrm{kg}m^{-3}$) the Monod constant. An alternative approach would be to incorporate maintenance metabolism, used in Buschulte (1992) and Cui et al. (1998):

(5) $$q_{O_{2}}\left(r\right)=\frac{\rho_{h}}{Y_{X/O_{2}}}\frac{c_{O_{2}}\left(r\right)-c_{O_{2},\mathrm{crit}}}{K_{\mathrm{XO}}+c_{O_{2}}\left(r\right)}{µ}_{\max}c_{h}\left(r\right)+m_{O_{2},\max}\rho_{h}\frac{c_{O_{2}}\left(r\right)}{K_{\mathrm{MO}}+c_{O_{2}}\left(r\right)}c_{h}\left(r\right),$$

where $m_{O_{2},\max}$ (${\mathrm{kgkg}}^{-1}h^{-1}$) is the maximum maintenance coefficient, $K_{\mathrm{MO}}({\mathrm{kgm}}^{-3})$ the Monod constant for maintenance and $c_{O_{2},\mathrm{crit}}({\mathrm{kgm}}^{-3})$, the critical concentration up to which biomass production occurs. Both approaches for describing the consumption term were tested in this study, and their suitability was assessed using the Akaike information criterion (AIC) (see Section 2.8.3). A summary of the parameter values required for the calculation is shown in Table 1.

**Table 1:** Parameter values from literature used in this study.

Parameter			Values		
Diffusion coefficient	$D_{O_{2},\mathrm{bulk}}$	Oxygen, 37°C	$8.7•10^{-6}$	$m^{2}h^{-1}$	Han and Bartels (1996), Jamnongwong et al. (2010)^a^
Diffusion hindrance factor	a	Mycelial networks	1.76	—	Schmideder et al. (2021)
Hyphal density (dry weight)	$\rho_{h}$	*Aspergillus niger NRRL‐3 and AB1.13*	150	${\mathrm{kgm}}^{-3}$	Rinas et al. (2005)
Oxygen Monod constant	$K_{\mathrm{XO}}$	*Gibberella fujikuroi strain*	$2.5∙10^{-5}$	${\mathrm{kgm}}^{-3}$	Escamilla Silva et al. (2001)
Maintenance Monod constant	$K_{\mathrm{MO}}$		$1.5{∙10}^{-5}$	${\mathrm{kgm}}^{-3}$	guessed
Yield coefficients for biomass	$Y_{X/O_{2}}$	*A. niger NRRL‐3 and AB1.13*	2.77	${\mathrm{kgkg}}^{-1}$	Rinas et al. (2005)
Critical oxygen concentration	$c_{O_{2},\mathrm{crit}}$		$1{∙10}^{-5}$	${\mathrm{kgm}}^{-3}$	Guessed
Maximum maintenance coefficient	$m_{O_{2},\max}$	*Aspergillus awamori*	$7.2∙10^{-3}$	${\mathrm{kgkg}}^{-1}h^{-1}$	Cui et al. (1998)
Maximum growth rate	${µ}_{\max}$	*A. niger* SKAn1015	0.16	$h^{-1}$	Wucherpfennig, Hestler and Krull (2011)
		*A. niger* MF22.4	0.24	$h^{-1}$	Kwon et al. (2013)

a Interpolation of the temperature dependence of Han and Bartels (1996) considering the influence of glucose, described in Jamnongwong et al. (2010).

It is presumed that the change in oxygen concentration is pseudo‐stationary since the growth processes and, thus, the change in the local hyphal fraction are much slower than the diffusion and consumption processes (Cui et al. 1998). To support this, characteristic times were estimated for these processes using Equations 1S to 3S, as detailed in the Supplementary Information. As can be seen in Supporting Information S1: Figure 1S, oxygen uptake occurs rapidly, typically within seconds. Diffusion processes into the pellet range from seconds to minutes, while the doubling time of the fungi takes approximately 3–4 h, depending on the maximal growth rate. Hence, the hyphal fraction can be assumed to be constant for the typical oxygen measurement duration.

#### 2.8.1 Numerical Solution

The method of lines was chosen to solve the 1D model (Equation 2). The discretization of the spatial direction using 100 grid points leads to a system of ordinary equation where the partial derivatives are approximated with a five‐point upwind scheme. The solution was computed using the MATLAB ODE solver ode15s, and the mass matrix was applied to set the boundary conditions. Due to the symmetry condition, a Neumann boundary condition was used inside the pellet ($\frac{\partial c_{O_{2}}\left(r=0\right)}{\partial r}=0$), while a Dirichlet boundary condition was used at the pellet border and kept constant at the maximum oxygen concentration ($c_{O_{2}}\left(r=r_{\max}\right)=c_{O_{2},\max}$). Assuming pseudo‐stationarity, the equilibrium oxygen concentration was aimed for. To achieve this, an initial concentration of $c_{O_{2},\max}$ was assigned at each point in the pellet at the beginning of the simulation, and the simulation was run until the concentration reached an equilibrium state. This was achieved within milliseconds (simulation time), as evidenced by preliminary trials that monitored concentration changes over time until no further changes were observed. To ensure stability, the simulation time was extended to 2 s and referred to the result as a steady‐state oxygen profile. The hyphal fraction from the CT data served as input data, which remained constant over the simulation period.

#### 2.8.2 Parameter Estimation

Various combinations of parameters were optimized (Supporting Information S1: Table 2S), while the remaining model parameters adhered to literature values. The significance of each parameter optimization for aligning with the experimental oxygen concentration was evaluated using the AIC (see Section 2.8.3), thus preventing overfitting. The optimization problem is formulated to minimize, with respect to parameters p, the sum of the squares of the residuals across n radial positions within the pellet. Residuals are defined as the differences between the simulated $y_{i,\mathrm{sim}}$ and measured $y_{i,\text{exp}}$ oxygen concentrations, assessed at each corresponding radial position i from 1 (at r=0) to n (at $r=r_{\max}$):

(6) $$\min_{p}\sum_{i=1}^{n}{\left(y_{i,\text{exp}}-y_{i,\mathrm{sim}}\right)}^{2}.$$

For this purpose, the MATLAB global optimizer Multistart was applied, testing 50 initial points. The optimization problem was calculated during the global optimization with MATLAB lsqnonlin and the trust‐region‐reflective algorithm. The Jacobi matrix was computed using complex step differentiation (CSD) to speed up the calculations. When used correctly, the CSD is more accurate than finite differences (Jayapragasam, Bideau, and Loulou 2018). The parameter optimization was conducted individually for each pellet. The goodness‐of‐fit was measured by the mean absolute error (MAE), where n represents all measured data points in a pellet:

(7) $$\mathrm{MAE}=\frac{1}{n}\sum_{i=1}^{n}\mathrm{abs}\left(y_{i,\text{exp}}-y_{i,\mathrm{sim}}\right).$$

#### 2.8.3 Model Selection

The AIC for small sample sizes of less than 40 (${\mathrm{AIC}}_{c}$) was applied to avoid overfitting and select the appropriate model by either considering (Equation 4) or neglecting maintenance metabolism (Equation 5).

(8) $${\mathrm{AIC}}_{c}=n\left[\ln\left(\frac{\mathrm{RSS}}{n}\right)\right]+2k+\frac{2k\left(k+1\right)}{n-k-1}.$$

Various parameters were tested while keeping the others constant at the values shown in Table 1. In Equation (8), RSS is the residual sum of squares, n the number of samples, and k the number of fitted parameters increased by one to include the variance as an additional parameter in least‐squares regression analysis. The best model is characterized by the smallest ${\mathrm{AIC}}_{c}$ value (Banks and Joyner 2017; Symonds and Moussalli 2011).

## 3 Results and Discussion

### 3.1 Cultivation and Data Generation

**Figure 3:**
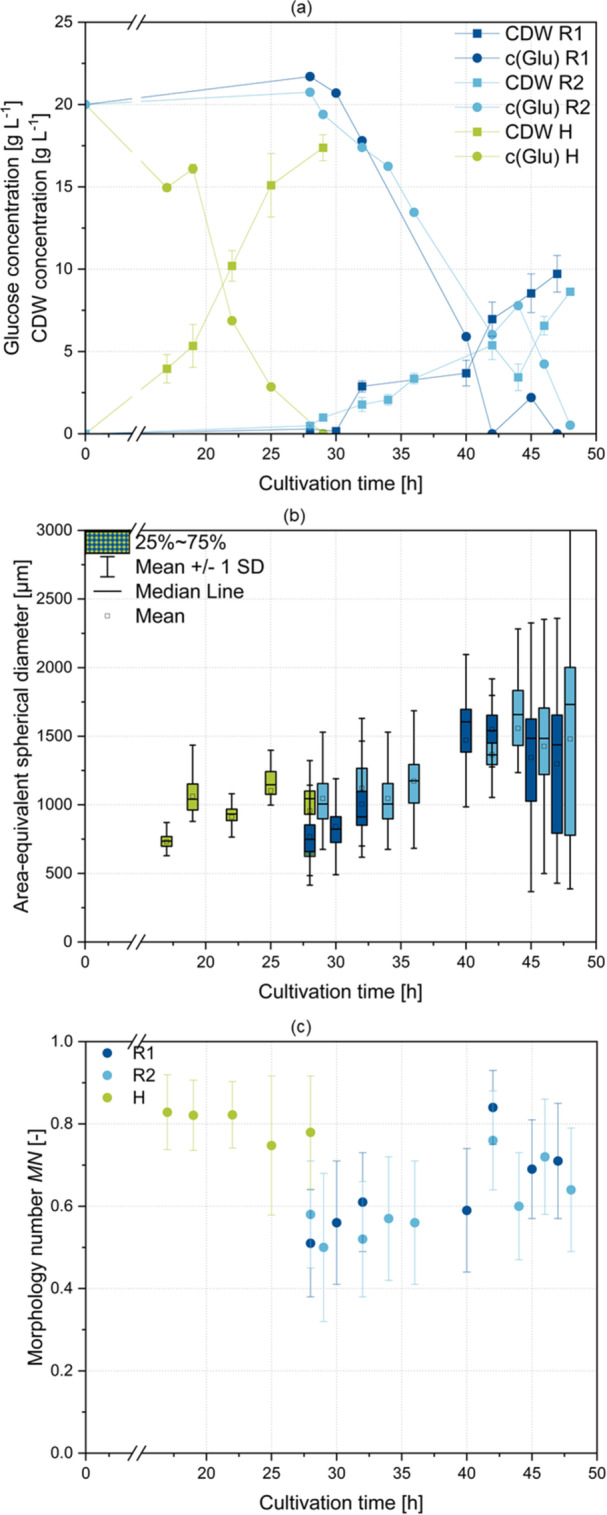
Summary of cultivation data for cultivation R1 (dark blue), R2 (light blue), and H (green). (a) Concentration of glucose (round) and cell dry weight (CDW) (square). (b) Course of area‐equivalent spherical diameter (AESD) derived from microscopic images. (c) Course of Morphology number (MN) derived from microscopic images.

In previous cultivations, the growth of both regular (R) and hyperbranching (H) *A. niger* strains were characterized to establish a timeframe for sampling and analysis of pellets. The limiting factor was obtaining pellets suitable for oxygen profiling, which required a minimum pellet diameter of approximately 500 µm to ensure pellet fixation in the flow tube. Additionally, glucose availability was crucial, as only pellets in the growth phase were to be analyzed. The cultivation results of the monitored main cultivations R1, R2, and H are presented in Figure 3.

Due to the rapid growth of hyperbranching cultivation H, the timeframe for analysis and sampling was set from 17 to 29 h of cultivation (Figure 3a). For cultivations R1 and R2, the timeframe was delayed and scheduled for 28–47 h and 28–49 h, respectively. For both strains, an initial increase in the area‐equivalent spherical diameter (AESD) can be observed until reaching a plateau value for the median at approximately 1000 µm for cultivation H and 1500 µm for R1 and R2 (Figure 3b). However, the MN of pellets originating from cultivation H displays a high value fluctuating around 0.8 (indicating relatively spherical pellets), while MN for R1 and R2 increases during the cultivation, reaching a value around 0.7 (Figure 3c).

**Figure 4:**
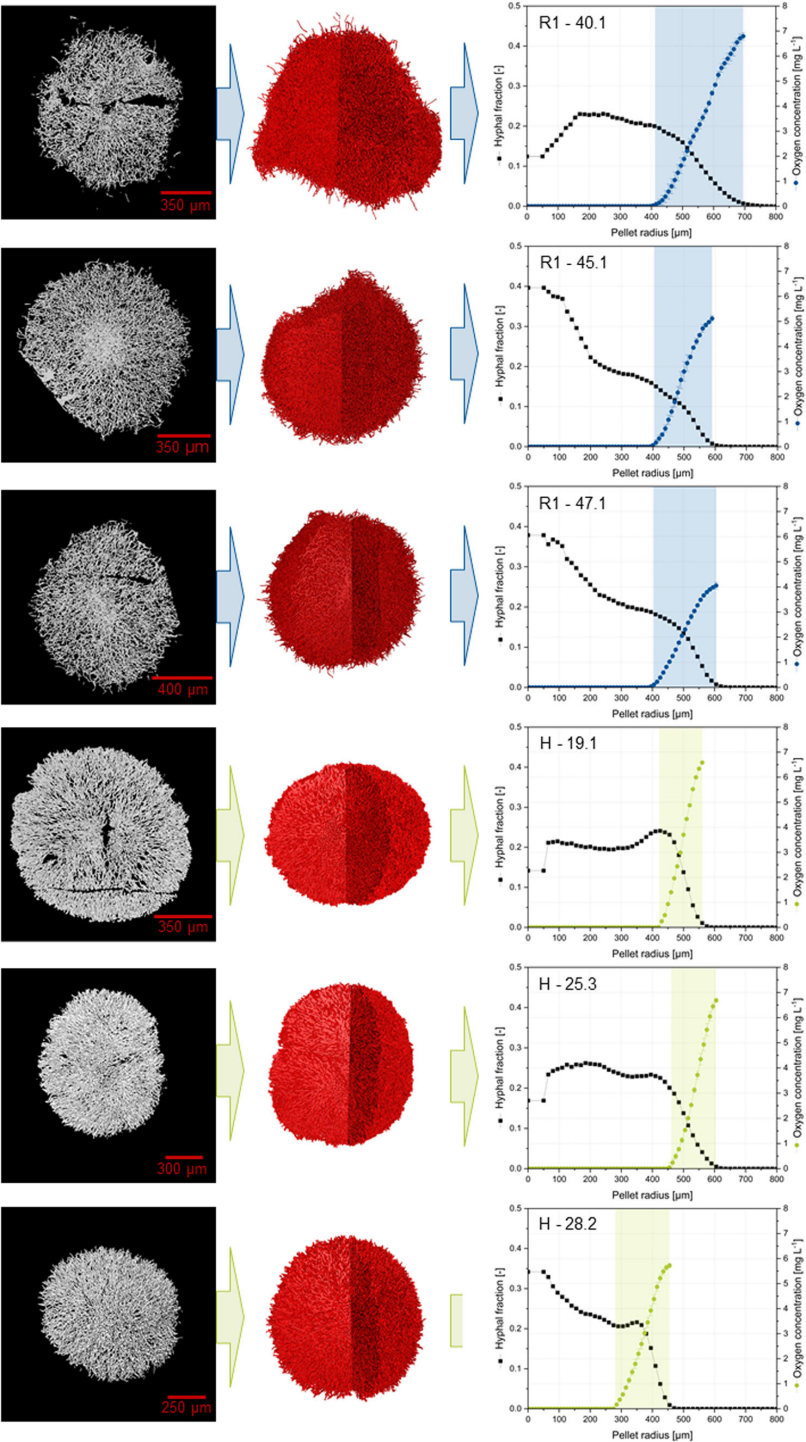
Data processing from 3D pellet images to radius‐resolved information. For each oxygen‐profiling pellet, 2D cross sections (left) of pellets generated using µCT are being assembled as 3D images (red pellet, middle) depicting the entire pellet. Structural data analysis provides radius‐resolved hyphal fractions (black plot on the right), which can be correlated with the measured oxygen concentration profile (blue plot for strain R, green for strain H). The plot label states the identification of single pellets (e.g., “H—28.2”: cultivation H, sample time 28 h, pellet number 2).

From each sample point, a minimum of three pellets were isolated, and oxygen profiling was subsequently conducted according to Figure 1. Following this, the same pellets were utilized for image analysis using µCT (Figure 2). Consequently, high‐resolution 2D cross‐sections and 3D whole‐pellet images were generated according to Schmideder, Barthel, Friedrich et al. (2019). The maximum radii and total hyphal volumes (overall volume of biomass) of the individual pellets are available in Supporting Information S1: Figure 2S. Figure 4 showcases six exemplarily selected pellets. For each visualized pellet, structural data, such as the radius‐resolved hyphal fraction, was noninvasively derived from the pellet interior and plotted in alignment with the oxygen concentrations obtained from the initial oxygen profiling. The hyphal fraction is plotted from the pellet mass center towards increasing pellet radii until defining the pellet border, at which the oxygen concentration represents 95% of the oxygen saturation concentration. Approaching the pellet center starting at the pellet border, the local oxygen concentration decreases gradually until reaching a point of oxygen depletion, confining the oxygen‐supplied layer. The slope of the oxygen profile is thereby dependent on several factors, such as the hyphal fraction and activity in the specific area. For instance, as the hyphal fraction appears higher for strain H, particularly in the outer region, the oxygen profile is also steeper. Detailed assessments of pellet images, hyphal fractions, and oxygen concentration profiles for all other pellets can be found in Supporting Information S1: Figures 3S−4S. In all profiles, a sharp decline in oxygen concentration is observed, with a relatively large central region showing zero oxygen concentration, except for sample R1 30.2 and samples H 17.3, H 22.1, and H 25.1. The latter three exceptions of strain H are considered unreliable due to pellet displacement during microelectrode penetration, and thus, these data are excluded from further analysis. For sample R1 30.2, additional evaluations were performed; however, these results should be interpreted cautiously, as the small pellet size and loose structure may have caused the suction method to distort the curve. All other data are considered reliable. Based on the comprehensive analysis of this data, the illustrated conclusions presented in the following sections have been derived, laying the groundwork for parameter estimation and modeling of oxygen concentrations along the pellet radius.

### 3.2 Analysis of Oxygen Supply and Hyphal Fraction

**Figure 5:**
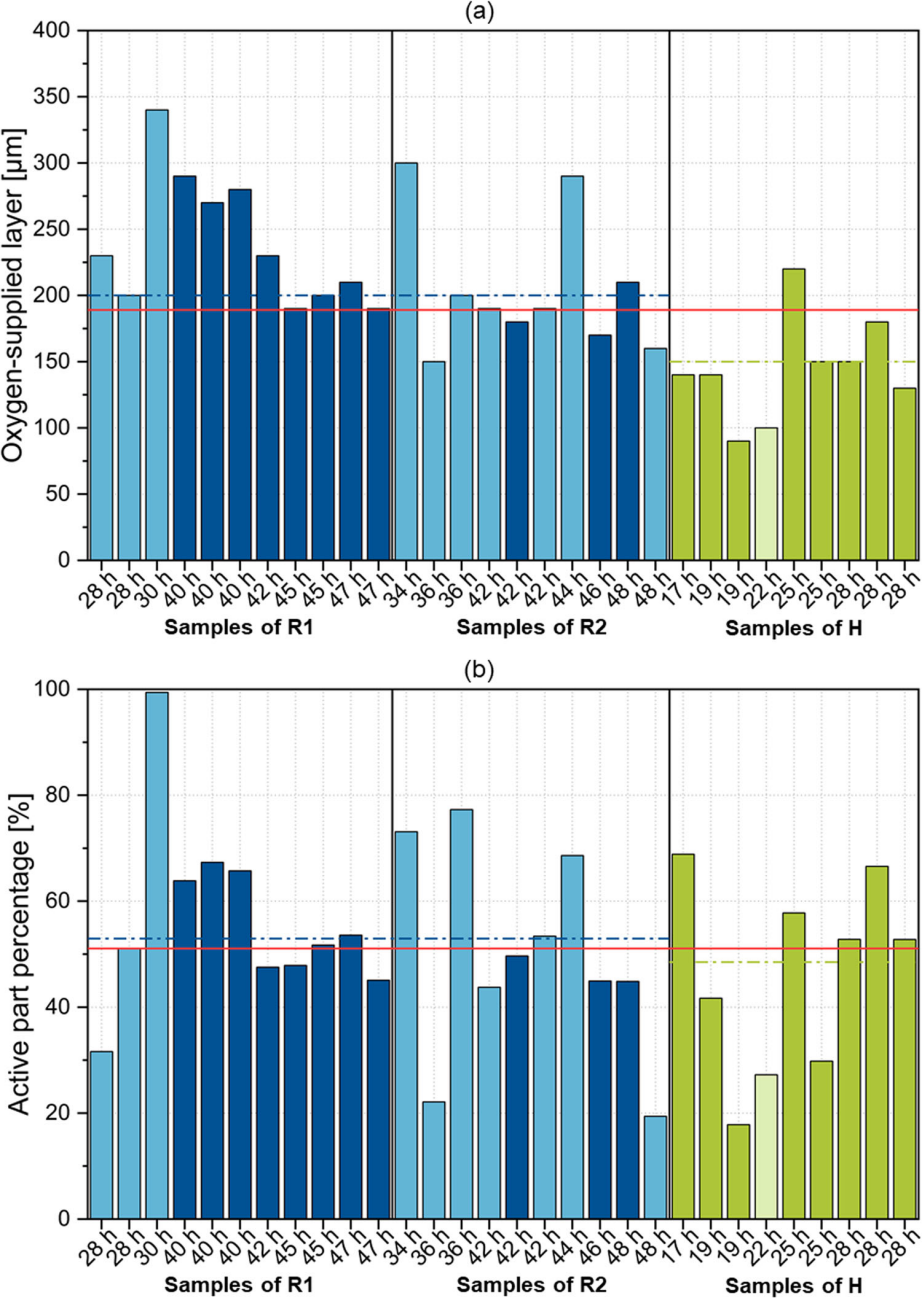
Oxygen supply in the pellet. (a) Thickness of the outer shell in which an oxygen concentration above zero is present. (b) Active part percentage (APP) calculated by determining the proportion of the hyphal volume within the supplied shell to the total hyphal volume in the pellet. Spherical (dark colored) and nonspherical (light colored) pellets are indicated. Mean value of all spherical pellets (red), originating from cultivation R1 and R2 (blue, dashed), and H (green, dashed) are highlighted.

**Figure 6:**
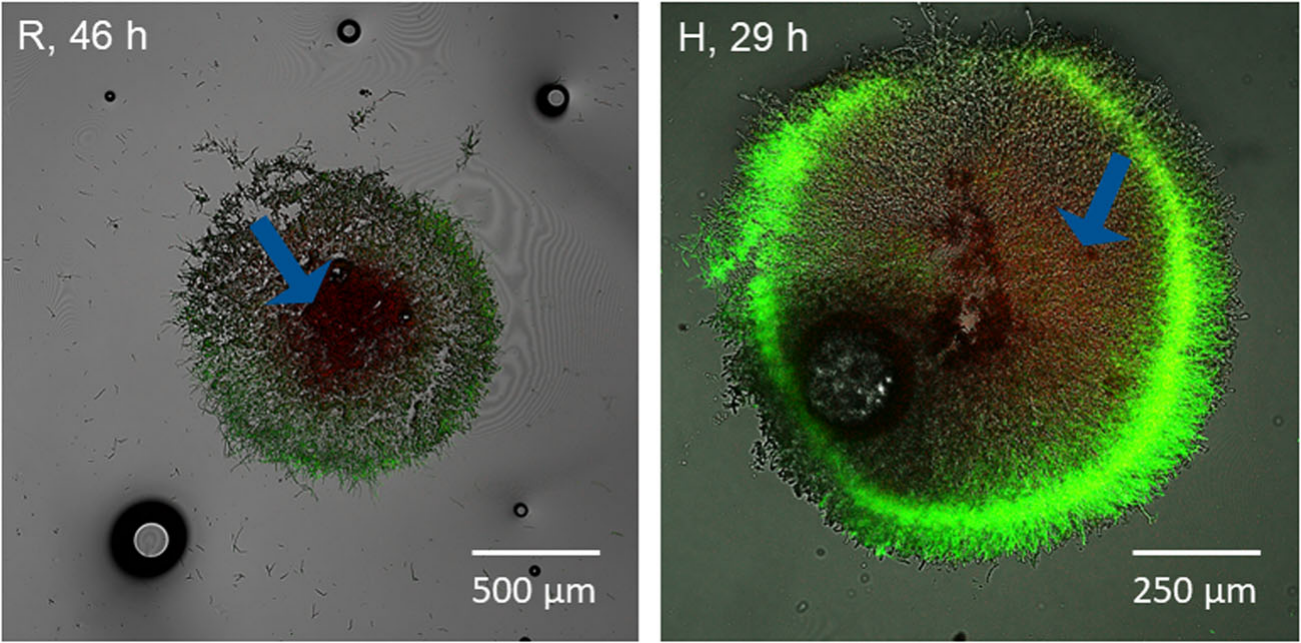
CLSM images for two exemplary pellets of strain R (46 h of cultivation) and strain H (29 h of cultivation). Staining was performed with SYTO9 (green fluorescence, living cells) and PI (red fluorescence, dead cells). Blue arrow indicates dead (red) areas.

Experimentally determined oxygen concentrations and hyphal fraction distributions derived from 3D pellet imaging were utilized to assess the oxygen supply to hyphae within the pellet. The one‐dimensional oxygen measurement and the method for calculating the hyphal fraction in spherical shells were designed for perfectly spherical pellets. Thus, the reliability of the analyses is highly affected by the sphericity of each pellet. Therefore, pellets were classified into spherical and nonspherical. Pellets were considered spherical when having a minimum axis ratio of 0.7 and a sphericity of 0.95. Consequently, only spherical pellets were considered for calculating mean values and drawing conclusions. Among the 30 measured pellets, 19 were spherical (11 for R and 8 for H). Axis ratio and sphericity data for all pellets are available in Supporting Information S1: Table 1S. In general, it can be stated that values calculated here better represent the total population of H due to the higher MN (Figure 3), as more spherical pellets exist. The measurements revealed that oxygen was only available in the outer regions (90–290 µm depth) of the pellet, with an average depth of 191 ± 53 µm (Figure 5a). Interestingly, an ANOVA analysis indicated significant differences (*p*‐value 0.0016) regarding the depth of the supplied pellet layer among the different strains, with a mean of 220 ± 42 µm for R and 150 ± 38 µm for H. Thereby, the APP varied from 18% to 69% (Figure 5b)**.** However, there was no significant difference (*p*‐value 0.88) in the APP between strain R and strain H, with a mean of 53% ± 9% for R and a mean of 49% ± 18% for H, indicating that penetration depth alone does not accurately reflect the fraction of supplied hyphae. Hille et al. (2005) determined penetration depths in *A. niger* AB1.13 using oxygen profiling to be approximately 250 µm for loose and 150 µm for compact pellets. These values correspond to the values obtained here. In other studies, hyphal activity in *A. niger* pellets was investigated using GFP co‐expression. Here, highly active regions were found to be only limited to the outer 75 µm (Driouch, Sommer, and Wittmann 2010) and 13–156 µm (Tegelaar et al. 2020) and are associated with diffusion limitations of oxygen and other nutrients. Furthermore, Tang et al. (2015) introduced the APP as a parameter to describe the influence of different stirrers on the activity of the pellets. The values reported in that study are significantly lower than those presented here, ranging from 12% to 20%, possibly due to imprecise calculations using average values for penetration depths and hyphal fraction determination via microscopy and centrifugation. To draw a comparison, live/dead CLSM imaging on exemplary pellets originating from identical cultivation conditions was performed (Figure 6). Clearly, for R, the active (green) area exceeds the oxygen‐supplied layer estimated by profiling. Also, strain H shows a rather unsteady fluorescence in the viable area. Thus, compared to the results of the oxygen profiling (volumetric calculation), calculating the active share of the pellets (area‐related estimation) based on CLSM showed an overestimation for the active (green) part. It was hypothesized that activity calculation in pellets conducted using oxygen profiling is more accurate than conventional CLSM, as external factors affecting the CLSM measurements, such as incubation and diffusion of dye and laser settings, can be excluded. Overall, a high APP in filamentous pellets is crucial for achieving high production efficiency (Gonciarz, Kowalska, and Bizukojc 2016; Schrinner et al. 2020). Therefore, it is a relevant factor to assess the potential for process improvement, such as through morphology engineering, which often aims to enhance the oxygen supply into pellets.

**Figure 7:**
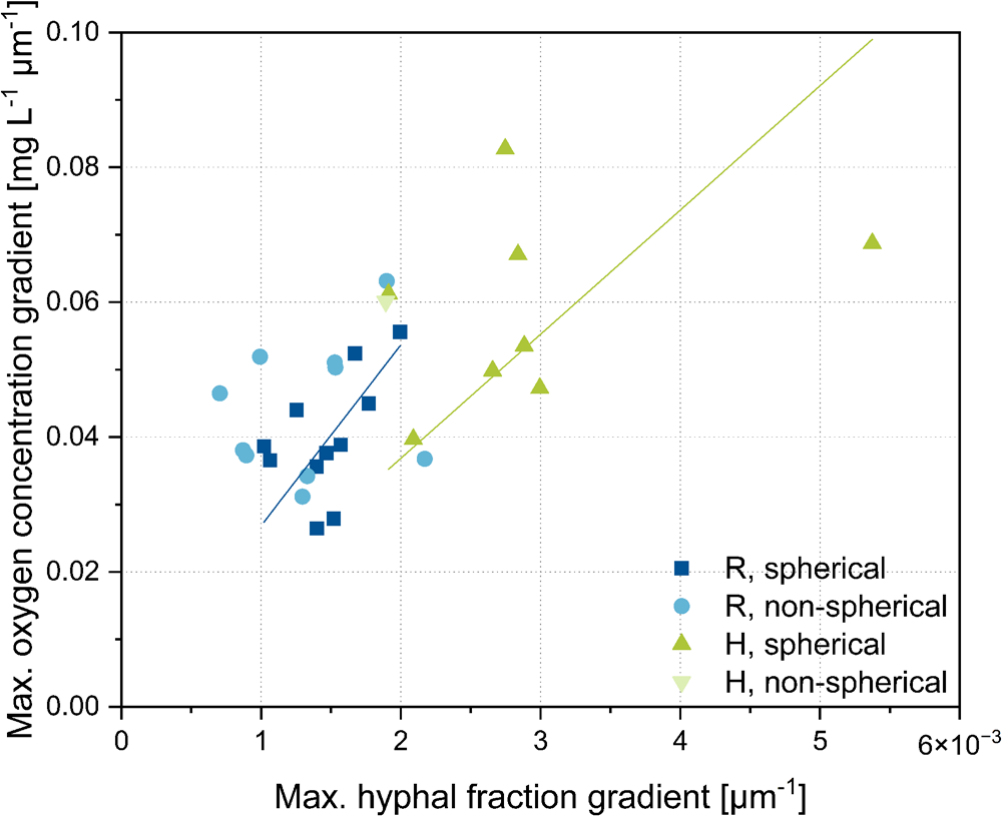
Correlation of maximum oxygen concentration gradient with maximum hyphal fraction gradient in oxygen supplied pellet area. Blue data represents strain R, green represents strain H. Spherical (dark colored) and nonspherical (light colored) pellets are indicated. Slopes for the linear fits of spherical pellets are 26.84 mg L^−1^ µm^−1^ for strain R (*R* ^2^ = 0.96335) and 18.42 mg L^−1^ µm^−1^ for strain H (*R* ^2^ = 0.89969).

To investigate the relationship between oxygen concentration profiles and hyphal fraction distributions, the maximum gradient in the oxygen curve and the maximum gradient in the hyphal fraction curve within the oxygen‐supplied region were plotted against each other for all spherical pellets (Figure 7)**.** Generally, strain H exhibits higher gradients than strain R, which aligns with the values of the oxygen‐supplied pellet layer. Accordingly, Hille et al. (2005) also calculated these, with the maximum gradients in the oxygen curves similar to ours, but the maximum gradients of the hyphal fraction were an order of magnitude too high. One possible reason for this could be the CLSM method used to determine the hyphal fraction. Despite the correlation between the maximum gradients, these scalar quantities are insufficient to infer oxygen availability in the pellet. Therefore, a simulation study using a diffusion‐reaction model was subsequently conducted to compute the oxygen concentration based on the hyphal fraction distribution.

### 3.3 Parameter Estimation and Simulation

**Figure 8:**
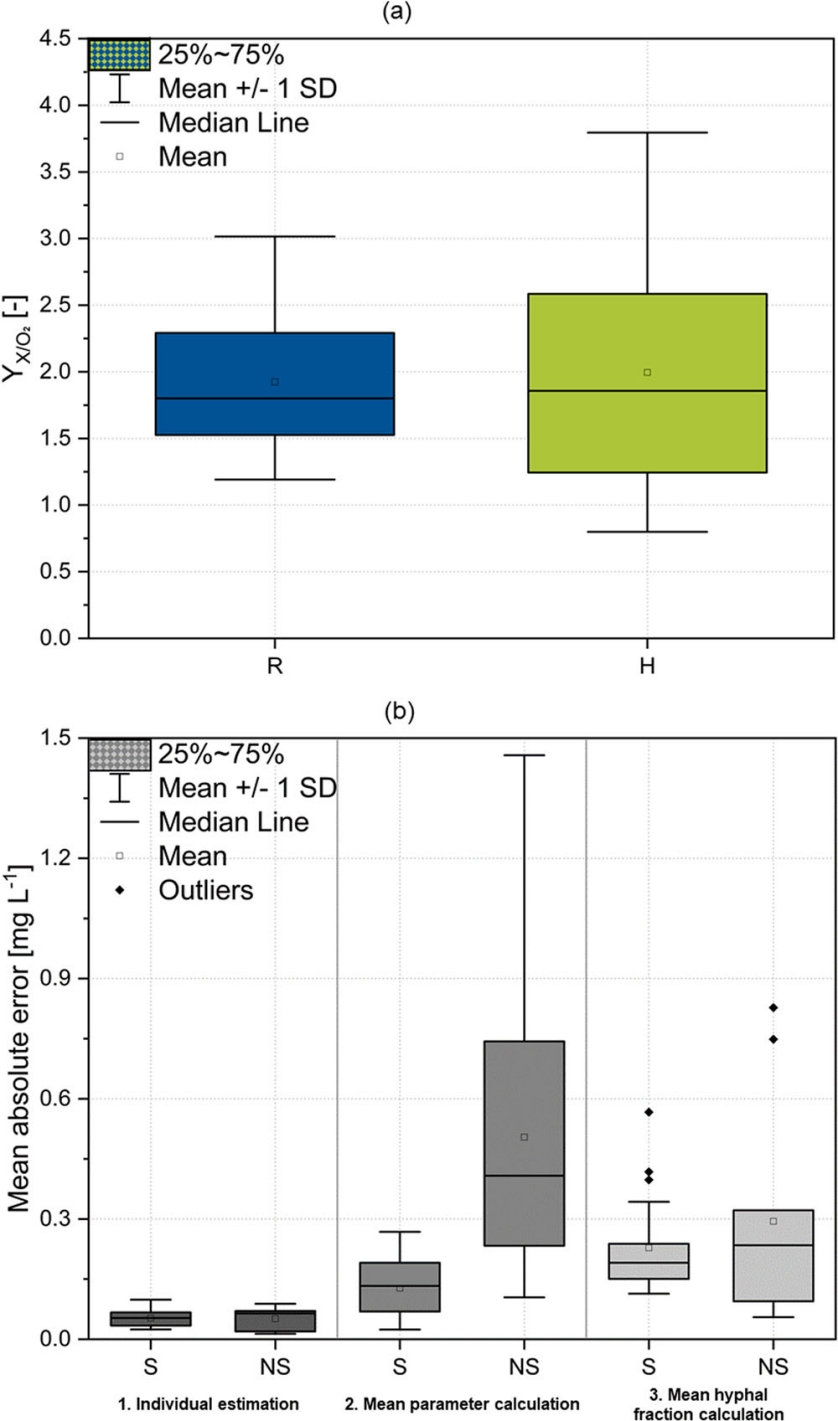
Yield coefficient $Y_{X/O_{2}}$ and mean absolute error (MAE) for all examined pellets. (a) $Y_{X/O_{2}}$ was calculated for individual pellets of strain R (blue) and strain H (green) to be 1.95. No significant differences can be determined between R (1.94) and H (1.99) (*p*‐value 0.84). (b) On the basis of parameter estimation, MAE for the oxygen concentration profile computation was conducted by: (1) using the pellet specific estimated parameter for calculating the oxygen concentration in individual pellets; (2) using all pellets' mean value for calculating the oxygen concentration in individual pellets; (3) using a constant hyphal fraction in the pellet, averaged over all pellets within each strain for calculating the oxygen concentration in individual pellets. MAE was calculated for spherical (S) and nonspherical (NS) pellets.

The aim of the following section is to find an appropriate model and estimate the necessary parameters to accurately predict the oxygen concentration based on the local hyphal fraction in the pellet. To accomplish this, a continuum‐scale model is utilized, where diffusion and reaction processes are locally computed. First, the AIC is used to decide whether the maintenance metabolism can be neglected and to define how many parameters in the model should be optimized without overfitting the data (Supporting Information S1: Table 2S). For this purpose, both the consumption coefficients $Y_{X/O_{2}}$and $m_{O_{2},\max}$, along with the Monod kinetic parameters ($K_{\mathrm{XO}},K_{\mathrm{MO}},$ $c_{O_{2},\mathrm{crit}}$) in Equations (4) and (5) are first optimized. The adjustment of a large number of parameters, in addition to the issue of overfitting, which is accounted for by the AIC as it relates the number of estimated parameters to the deviation of the model solution from the experimental data, also increases the likelihood that parameter values cannot be uniquely determined due to the complexity of the parameter estimation problem. This occurs because combinations of parameters may lead to similar results, thus similarly well representing the experimental data. This causes significant variability in parameter values across individual parameter estimations (see: Spreading in all pellets; Supporting Information S1: Table S2). In our analysis, we examined the potential impact of adjusting solely the consumption kinetics parameters on the model solution, as well as the effectiveness of solely adjusting the Monod kinetic parameters. Throughout this process, the AIC was consistently utilized to assess and verify the quality of the estimation. The model exhibiting the lowest ${\mathrm{AIC}}_{c}$ was the one where maintenance metabolism was not considered separately (Equation 4) and only the yield coefficient $Y_{X/O_{2}}$ was optimized, while the remaining parameters matched the literature values from Table 1. It is important to note that this applies to the present data set. For a data set in which oxygen concentrations exhibit a flatter profile and a larger area showing low concentrations above zero, indicating no biomass growth but maintenance metabolism, a different model may be more appropriate. In the pellets used for oxygen profiling, where no factors other than oxygen limitations are present, oxygen is rapidly consumed by the outer pellet layer, dropping to zero and resulting in only a small area in the pellet suitable for parameter identification (Figure 4). Derived from the experimental data, $Y_{X/O_{2}}$was individually estimated for each pellet (Supporting Information S1: Figure 5S). No trends in the estimated $Y_{X/O_{2}}$ values regarding the cultivation time could be identified and an ANOVA demonstrated that there was no significant difference between the two strains (*p*‐value 0.84). However, a spreading in the estimated yield coefficients can be observed, which is notably larger for strain H (Figure 8a). This could be attributed to the activity of the pellets, which might also vary to a higher degree in strain H. The average value of $Y_{X/O_{2}}$for all pellets is 1.95 (± 0.72), which is in the same order of magnitude as the literature values for *A. niger*: 2.77 (Rinas et al. 2005) and 1 (Cui et al. 1998). Thereby, higher $Y_{X/O_{2}}$ values represent improved metabolic efficiency for biomass production. In the model, it is assumed that under equal oxygen supply, biomass increases identically, or at optimal supply, it grows at maximum specific growth rate *µ*_max_. A higher $Y_{X/O_{2}}$ indicates that less oxygen is required as compared to a lower one. It is important to acknowledge that, in reality, metabolic processes for the formation of primary or secondary products that consume oxygen also occur. However, they have not been accounted for in this model. The study specifically focuses on the early cultivation phases, where oxygen consumption is predominantly associated with biomass production. Additionally, incorporating these metabolic processes would necessitate extensive data on product‐specific rates.

**Figure 9:**
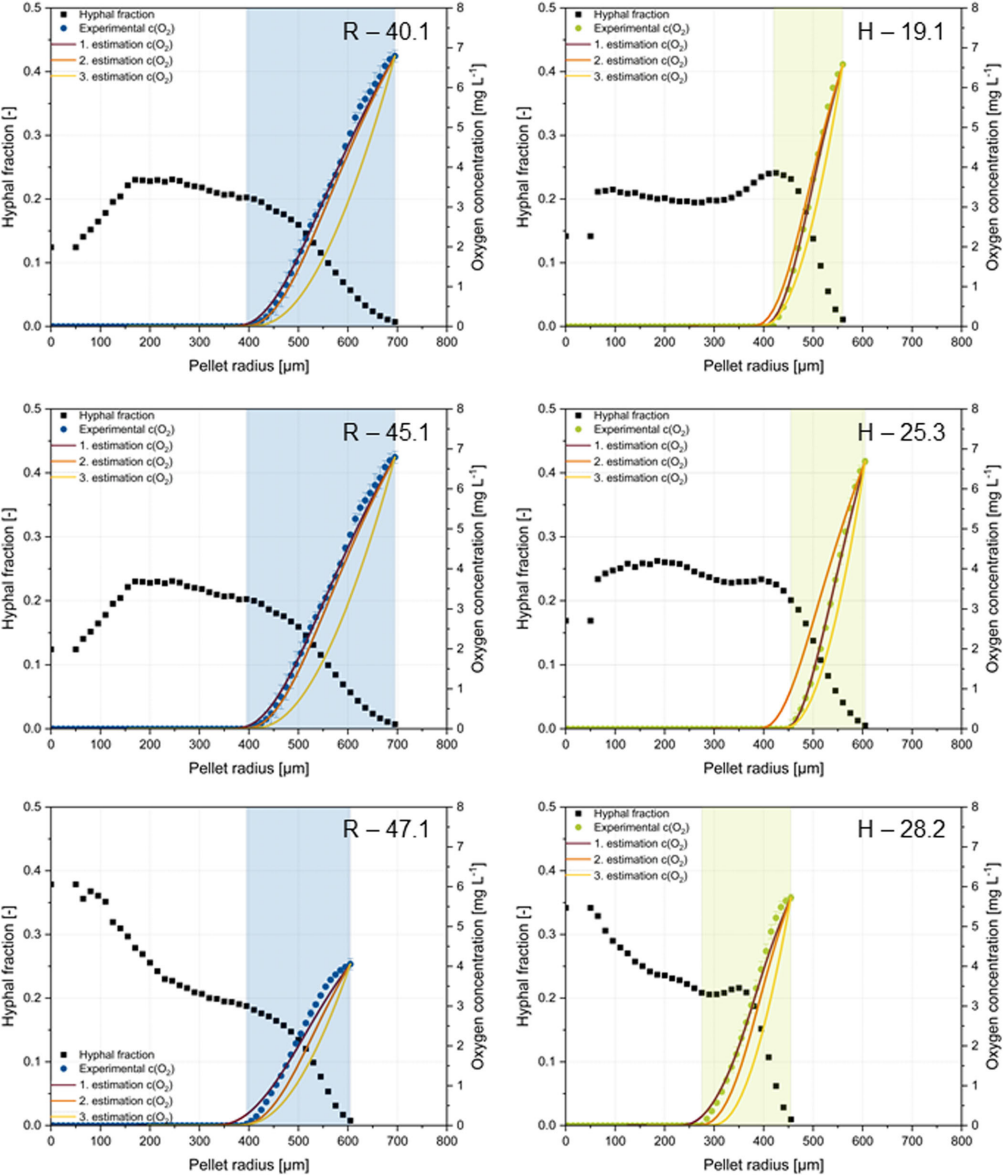
Pellet data derived from 3D imaging, oxygen profiling and oxygen simulation for three exemplary pellets of strain R and strain H. For each oxygen‐profiling pellet structural data analysis provides radius‐resolved hyphal fractions (black plot), which can be correlated with the measured oxygen concentration profile (blue plot for strain R, green for strain H). On the basis of parameter estimation, the oxygen concentration profile was computed by: (1) using the pellet specific estimated parameter for calculating the oxygen concentration in individual pellets (red); (2) using all pellets´ mean value for calculating the oxygen concentration in individual pellets (orange); (3) using a constant hyphal fraction in the pellet, averaged over all pellets within each strain for calculating the oxygen concentration in individual pellets (yellow). The plot label states the identification of single pellets (e.g., “H−8.2”: cultivation H, sample time 28 h, pellet number 2).

To assess the goodness of the applied fit, the MAE was calculated (Figure 8b). First, it was calculated for the individually estimated values (1. Individual estimation). The fit goodness was also evaluated by aligning with the computed curve using the mean parameter 1.95 in each pellet (2. Mean parameter calculation). This provided an estimate of how well the oxygen concentrations in pellets can be predicted based on available information on the local hyphal fraction, for example, from µCT measurements, but in the absence of experimentally determined oxygen concentrations and individual parameter estimates. Consequently, when using the average parameter for the simulation, the biological variability between pellets is neglected, which leads to an error that can be determined via the goodness of fit. In a third calculation of MAE, a constant hyphal fraction in the pellet averaged over all pellets within each strain was employed alongside the mean parameter (3. Mean hyphal fraction calculation) to estimate how accurately oxygen concentration can be predicted when no 3D morphological data is available, but only the pellet diameter. Simulation results for the showcased pellets in Figure 4 are depicted in Figure 9. The simulation of all other pellets can be found in Supporting Information S1: Figure 6S. With individual estimation, an average deviation from experimental values of 0.05 mg L^−1^ was observed. This discrepancy falls within the range of error bars in the experimental data. The good fit suggests that the developed model has hardly any conceptual or systematic errors and is well‐suited for the simulation. However, the mean parameter calculation performs poorly for nonspherical pellets, indicating an inadequate description of the hyphal fraction in the spherical shell around the mass center. Interestingly, the fit for nonspherical pellets improves with the mean hyphal fraction calculation, as it avoids underestimating the hyphal fraction in the outer pellet region. The enhancement achieved by locally resolved hyphal fraction calculation using the mean parameter over mean hyphal fraction calculation is approximately 0.1 mg L^−1^ per measurement point in the pellet (mean parameter calculation spherical MAE ~ 0.12 mg L^−1^, mean hyphal fraction calculation spherical MAE ~ 0.22 mg L^−1^) (Figure 8b).

The distribution of hyphae within the pellet enhanced the applicability of the model, representing an improvement over previous models, such as that proposed by Rinas et al. (2005), which assumed constant values for the hyphal fraction. Nevertheless, our modeling approach still faces limitations due to the inability to accurately represent diverse pellet shapes. To address this issue, future models are proposed to directly integrate 3D structures from µCT data as inputs. This approach would improve model accuracy by eliminating the need to evaluate hyphal fractions in spherical shells. However, this method will likely increase computational demands, as it would necessitate using Finite Element or Finite Volume methods for analysis. Additionally, it would be interesting to investigate the causes behind the significant parameter variation across pellets, likely due to variability in pellet activity. Furthermore, considering morphological information, such as tip distributions within the pellet and hyphal lengths as employed in Buschulte's continuum approach (Buschulte 1992), could potentially improve the modeling of oxygen concentration.

## 4 Conclusion

This study successfully unifies three challenging disciplines in pellet characterization for the first time: structural elucidation through 3D imaging, oxygen supply analysis with microelectrode measurements, and the simulation of the oxygen supply based on estimated parameters. A workflow was established, consisting of cultivating the regular branching *A. niger* strain SKAn1015 (R) and the hyperbranching strain MF22.4 (H), isolating individual pellets for oxygen profiling in a flow cell, transferring those pellets to perform µCT imaging, and subsequently conducting image processing. The resulting hyphal fractions and oxygen concentration profiles formed the basis to discover a communality between both strains with a similar APP (51%) and an estimated yield coefficient $Y_{X/O_{2}}$ (1.95). These similarities are remarkable considering the genetic differences between the strains, particularly the hyperbranching modifications in strain H, and their different cultivation conditions. This suggests that intrinsic transport processes through the hyphal structure play a crucial role despite these genetic and environmental variations. However, discrepancies arose in the thickness of the oxygen‐supplied pellet layer (200 µm for strain R and 150 µm for strain H), attributed to differing hyphal fractions within the outer pellet region.

To close the knowledge gap in description of oxygen consumption and biomass formation terms in pellets, the derived data was utilized for simulation of the oxygen concentration based on a mechanistic model. The oxygen simulation was initially conducted using individually estimated parameters for each pellet to assess the model's representation of the data and to examine the variance across all pellets. This method indicated that by adjusting the yield coefficient $Y_{X/O_{2}}$alone, a good fit of the data could be achieved, although variations in the estimated values were observed, possibly due to underlying biological variability among the samples. To further investigate the predictive capabilities of the model, the goodness of fit was assessed using an average parameter value estimated across all pellets. This strategy was aimed at evaluating the potential for future research to predict oxygen supply based on locally resolved hyphal fraction data. Given that such data may not always be available or was not measured in earlier studies, the prediction of oxygen concentration was additionally compared using mean densities.

The model developed in this study elucidates the relationship between hyphal distribution and oxygen supply within pellets. It can advance our understanding of fungal pellet morphology development, particularly considering the available oxygen within the pellet. Furthermore, this information can be used to optimize pellet structures, ensuring optimal oxygen supply for specific production goals. Additionally, these insights can also be utilized to model and analyze the entire cultivation process, providing a comprehensive framework for understanding and optimizing filamentous fungal growth at the macroscale. Thereby, heterogeneous pellet structures with different supplies in the pellet, as well as varying oxygen concentrations in the media in different zones within the reactor, can be considered.

## Author Contributions

Charlotte Deffur and Anna Dinius did the conception and design of the study. Charlotte Deffur and Anna Dinius wrote the manuscript, which was edited and approved by all authors. All authors interpreted the data. Heiko Briesen and Rainer Krull supervised the study. Julian Pagel and Anna Dinius cultivated filamentous fungi, conducted oxygen profiling and prepared pellets for microcomputed tomography (SR‐µ‐CT) measurements. Charlotte Deffur performed µ‐CT measurements of pellets. Charlotte Deffur reconstructed the image data. Henri Müller and Stefan Schmideder created the code for image analysis. Charlotte Deffur and Julian Pagel performed the image and data processing. Charlotte Deffur and Julian Pagel created the code for modeling the data. Anna Dinius creed the figures.

## Conflicts of Interest

The authors declare no conflicts of interest.

## Supporting information

Supporting information.

## Data Availability

The data that support the findings of this study are available from the corresponding author upon reasonable request.

## References

[bit28874-bib-0001] Banks, H. T. , and M. L. Joyner . 2017. “AIC Under the Framework of Least Squares Estimation.” Applied Mathematics Letters 74, no. 3: 33–45. 10.1016/j.aml.2017.05.005.

[bit28874-bib-0002] Böl, M. , K. Schrinner , S. Tesche , and R. Krull . 2021. “Challenges of Influencing Cellular Morphology by Morphology Engineering Techniques and Mechanical Induced Stress on Filamentous Pellet Systems‐A Critical Review.” Engineering in Life Sciences 21, no. 3–4: 51–67. 10.1002/elsc.202000060. 33716605 PMC7923580

[bit28874-bib-0003] Buschulte, T. C. 1992. “Mathematische Modellbildung und Simulation von Zellwachstum, Stofftransport und Stoffwechsel in Pellets aus Streptomyceten. (translated: Mathematical Modeling and Simulation of Cell Growth, Mass Transport and Metabolism in Streptomyces pellets.).” PhD thesis., Stuttgart: Universität Stuttgart.

[bit28874-bib-0004] Celler, K. , C. Picioreanu , M. C. M. van Loosdrecht , and G. P. van Wezel . 2012. “Structured Morphological Modeling as a Framework for Rational Strain Design of *Streptomyces* Species.” Antonie Van Leeuwenhoek 102, no. 3: 409–423. 10.1007/s10482-012-9760-9. 22718122 PMC3456926

[bit28874-bib-0005] Cronenberg, C. C. H. , S. P. P. Ottengraf , J. C. Heuvel , et al. 1994. “Influence of Age and Structure of *Pencillium chrysogenum* Pellets on the Internal Concentration Profiles.” Bioprocess Engineering 10, no. 5–6: 209–216. 10.1007/BF00369531.

[bit28874-bib-0006] Cui, Y. Q. , W. J. Okkerse , R. G. J. M. van der Lans , and K. C. A. M. Luyben . 1998. “Modeling and Measurements of Fungal Growth and Morphology in Submerged Fermentations.” Biotechnology and Bioengineering 60, no. 2: 216–229. 10.1002/(sici)1097-0290(19981020)60:2<216::aid-bit9>3.0.co;2-q. 10099423

[bit28874-bib-0007] Dinius, A. , Z. J. Kozanecka , K. P. Hoffmann , and R. Krull . 2024. “Intensification of Bioprocesses With Filamentous Microorganisms.” Physical Sciences Reviews 9, no. 2: 777–823. 10.1515/psr-2022-0112.

[bit28874-bib-0008] Dinius, A. , H. Müller , D. Kellhammer , et al. 2024. “3D Imaging and Analysis to Unveil the Impact of Microparticles on the Pellet Morphology of Filamentous Fungi.” Biotechnology and Bioengineering 121: 3128–3143. 10.1002/bit.28788. 38943490

[bit28874-bib-0009] Dinius, A. , K. Schrinner , M. Schrader , et al. 2023. “Morphology Engineering for Novel Antibiotics: Effect of Glass Microparticles and Soy Lecithin on Rebeccamycin Production and Cellular Morphology of Filamentous Actinomycete *Lentzea aerocolonigenes* .” Frontiers in Bioengineering and Biotechnology 11: 1171055. 10.3389/fbioe.2023.1171055. 37091334 PMC10116066

[bit28874-bib-0010] Driouch, H. , B. Sommer , and C. Wittmann . 2010. “Morphology Engineering of *Aspergillus niger* for Improved Enzyme Production.” Biotechnology and Bioengineering 105, no. 6: 1058–1068. 10.1002/bit.22614. 19953678

[bit28874-bib-0011] Emerson, S. 1950. “The Growth Phase in Neurospora Corresponding to the Logarithmic Phase in Unicellular Organisms.” Journal of Bacteriology 60, no. 3: 221–223. 10.1128/jb.60.3.221-223.1950. 14774340 PMC385870

[bit28874-bib-0012] Escamilla Silva, E. M. , G. F. Gutierrez , L. Dendooven , H. Jimenez , and J. A. Ochoa‐Tapia . 2001. “A Method to Evaluate the Isothermal Effectiveness Factor for Dynamic Oxygen Into Mycelial Pellets in Submerged Cultures.” Biotechnology Progress 17, no. 1: 95–103. 10.1021/bp0001361. 11170486

[bit28874-bib-0013] Fiedler, M. R. M. , L. Barthel , C. Kubisch , C. Nai , and V. Meyer . 2018. “Construction of an Improved *Aspergillus niger* Platform for Enhanced Glucoamylase Secretion.” Microbial Cell Factories 17, no. 1: 95. 10.1186/s12934-018-0941-8. 29908567 PMC6004097

[bit28874-bib-0014] Gonciarz, J. , A. Kowalska , and M. Bizukojc . 2016. “Application of Microparticle‐Enhanced Cultivation to Increase the Access of Oxygen to *Aspergillus terreus* ATCC 20542 Mycelium and Intensify Lovastatin Biosynthesis in Batch and Continuous Fed‐Batch Stirred Tank Bioreactors.” Biochemical Engineering Journal 109: 178–188. 10.1016/j.bej.2016.01.017.

[bit28874-bib-0015] Han, P. , and D. M. Bartels . 1996. “Temperature Dependence of Oxygen Diffusion in H_2_O and D_2_O.” The Journal of Physical Chemistry 100, no. 13: 5597–5602. 10.1021/jp952903y.

[bit28874-bib-0016] Hille, A. , T. R. Neu , D. C. Hempel , and H. Horn . 2005. “Oxygen Profiles and Biomass Distribution in Biopellets of *Aspergillus niger* .” Biotechnology and Bioengineering 92, no. 5: 614–623. 10.1002/bit.20628. 16136592

[bit28874-bib-0017] Hille, A. , T. R. Neu , D. C. Hempel , and H. Horn . 2009. “Effective Diffusivities and Mass Fluxes in Fungal Biopellets.” Biotechnology and Bioengineering 103, no. 6: 1202–1213. 10.1002/bit.22351. 19422038

[bit28874-bib-0018] Jamnongwong, M. , K. Loubiere , N. Dietrich , and G. Hébrard . 2010. “Experimental Study of Oxygen Diffusion Coefficients in Clean Water Containing Salt, Glucose or Surfactant: Consequences on the Liquid‐Side Mass Transfer Coefficients.” Chemical Engineering Journal 165, no. 3: 758–768. 10.1016/j.cej.2010.09.040.

[bit28874-bib-0019] Jayapragasam, P. , P. L. Bideau , and T. Loulou . 2018. “Computing Sensitivity Coefficients by Using Complex Differentiation: Application to Heat Conduction Problem.” Numerical Heat Transfer, Part B: Fundamentals 74, no. 5: 729–745. 10.1080/10407790.2019.1580047.

[bit28874-bib-0020] Krull, R. , T. Wucherpfennig , M. E. Esfandabadi , et al. 2013. “Characterization and Control of Fungal Morphology for Improved Production Performance in Biotechnology.” Journal of Biotechnology 163, no. 2: 112–123. 10.1016/j.jbiotec.2012.06.024. 22771505

[bit28874-bib-0021] Kwon, M. J. , B. M. Nitsche , M. Arentshorst , T. R. Jørgensen , A. F. J. Ram , and V. Meyer . 2013. “The Transcriptomic Signature of Raca Activation and Inactivation Provides New Insights Into the Morphogenetic Network of *Aspergillus niger* .” PLoS ONE 8, no. 7: e68946. 10.1371/journal.pone.0068946. 23894378 PMC3722221

[bit28874-bib-0022] Laible, A. R. , A. Dinius , M. Schrader , et al. 2021. “Effects and Interactions of Metal Oxides in Microparticle‐Enhanced Cultivation of Filamentous Microorganisms.” Engineering in Life Sciences 22, no. 12: 725–743. 10.1002/elsc.202100075. 36514528 PMC9731605

[bit28874-bib-0023] Lin, P.‐J. , A. Scholz , and R. Krull . 2010. “Effect of Volumetric Power Input by Aeration and Agitation on Pellet Morphology and Product Formation of *Aspergillus niger* .” Biochemical Engineering Journal 49, no. 2: 213–220. 10.1016/j.bej.2009.12.016.

[bit28874-bib-0024] Marshall, K. C. , and M. Alexander . 1960. “Growth Characteristics of Fungi and Actinomycetes.” Journal of Bacteriology 80, no. 3: 412–416. 10.1128/jb.80.3.412-416.1960. 13767223 PMC278880

[bit28874-bib-0025] Meyer, V. , T. Cairns , L. Barthel , et al. 2021. “Understanding and Controlling Filamentous Growth of Fungal Cell Factories: Novel Tools and Opportunities for Targeted Morphology Engineering.” Fungal Biology and Biotechnology 8, no. 1: 8. 10.1186/s40694-021-00115-6. 34425914 PMC8383395

[bit28874-bib-0026] Meyerhoff, J. , V. Tiller , and K.‐H. Bellgardt . 1995. “Two Mathematical Models for the Development of a Single Microbial Pellet.” Bioprocess Engineering 12, no. 6: 305–313. 10.1007/BF00369507.

[bit28874-bib-0027] Müller, H. , C. Deffur , S. Schmideder , et al. 2023. “Synchrotron Radiation‐Based Microcomputed Tomography for Three‐Dimensional Growth Analysis of *Aspergillus niger* Pellets.” Biotechnology and Bioengineering 120, no. 11: 3244–3260. 10.1002/bit.28506. 37475650

[bit28874-bib-0028] Pirt, S. J. 1966. “A Theory of the Mode of Growth of Fungi in the Form of Pellets in Submerged Culture.” Proceedings of the Royal Society of London. Series B, Biological Sciences 166, no. 1004: 369–373. 10.1098/rspb.1966.0105. 4382769

[bit28874-bib-0029] Rinas, U. , H. El‐Enshasy , M. Emmler , A. Hille , D. C. Hempel , and H. Horn . 2005. “Model‐Based Prediction of Substrate Conversion and Protein Synthesis and Excretion in Recombinant *Aspergillus niger* Biopellets.” Chemical Engineering Science 60, no. 10: 2729–2739. 10.1016/j.ces.2004.12.020.

[bit28874-bib-0030] Schmideder, S. , L. Barthel , T. Friedrich , et al. 2019. “An X‐Ray Microtomography‐Based Method for Detailed Analysis of the Three‐Dimensional Morphology of Fungal Pellets.” Biotechnology and Bioengineering 116, no. 6: 1355–1365. 10.1002/bit.26956. 30768872

[bit28874-bib-0031] Schmideder, S. , L. Barthel , H. Müller , V. Meyer , and H. Briesen . 2019. “From Three‐Dimensional Morphology to Effective Diffusivity in Filamentous Fungal Pellets.” Biotechnology and Bioengineering 116, no. 12: 3360–3371. 10.1002/bit.27166. 31508806

[bit28874-bib-0032] Schmideder, S. , H. Müller , L. Barthel , et al. 2021. “Universal Law for Diffusive Mass Transport Through Mycelial Networks.” Biotechnology and Bioengineering 118, no. 2: 930–943. 10.1002/bit.27622. 33169831

[bit28874-bib-0033] Schrinner, K. , L. Veiter , S. Schmideder , et al. 2020. “Morphological and Physiological Characterization of Filamentous *Lentzea aerocolonigenes*: Comparison of Biopellets by Microscopy and Flow Cytometry.” PLoS One 15, no. 6: e0234125. 10.1371/journal.pone.0234125. 32492063 PMC7269266

[bit28874-bib-0034] Stocks, S. M. 2004. “Mechanism and Use of the Commercially Available Viability Stain, Baclight.” Cytometry Part A. 61, no. 2: 189–195. 10.1002/cyto.a.20069. 15382024

[bit28874-bib-0035] Symonds, M. R. E. , and A. Moussalli . 2011. “A Brief Guide to Model Selection, Multimodel Inference and Model Averaging in Behavioural Ecology Using Akaike's Information Criterion.” Behavioral Ecology and Sociobiology 65, no. 1: 13–21. 10.1007/s00265-010-1037-6.

[bit28874-bib-0036] Tang, W. , A. Pan , H. Lu , et al. 2015. “Improvement of Glucoamylase Production Using Axial Impellers With Low Power Consumption and Homogeneous Mass Transfer.” Biochemical Engineering Journal 99: 167–176. 10.1016/j.bej.2015.03.025.

[bit28874-bib-0037] Tegelaar, M. , D. Aerts , W. R. Teertstra , and H. A. B. Wösten . 2020. “Spatial Induction of Genes Encoding Secreted Proteins in Micro‐Colonies of *Aspergillus niger* .” Scientific Reports 10, no. 1: 1536. 10.1038/s41598-020-58535-0. 32001779 PMC6992626

[bit28874-bib-0038] Veiter, L. , V. Rajamanickam , and C. Herwig . 2018. “The Filamentous Fungal Pellet‐Relationship Between Morphology and Productivity.” Applied Microbiology and Biotechnology 102, no. 7: 2997–3006. 10.1007/s00253-018-8818-7. 29473099 PMC5852183

[bit28874-bib-0039] Walisko, R. , J. Moench‐Tegeder , J. Blotenberg , T. Wucherpfennig , and R. Krull . 2015. “The Taming of the Shrew‐Controlling the Morphology of Filamentous Eukaryotic and Prokaryotic Microorganisms.” Advances in Biochemical Engineering/Biotechnology 149: 1–27. 10.1007/10_2015_322. 25796624

[bit28874-bib-0040] Wittier, R. , H. Baumgartl , D. W. Lübbers , and K. Schügerl . 1986. “Investigations of Oxygen Transfer into *Penicillium chrysogenum* Pellets by Microprobe Measurements.” Biotechnology and Bioengineering 28, no. 7: 1024–1036. 10.1002/bit.260280713. 18555424

[bit28874-bib-0041] Wucherpfennig, T. , T. Hestler , and R. Krull . 2011. “Morphology Engineering ‐ Osmolality and Its Effect on *Aspergillus niger* Morphology and Productivity.” Microbial Cell Factories 10: 58. 10.1186/1475-2859-10-58. 21801352 PMC3178489

[bit28874-bib-0042] Wucherpfennig, T. , A. Lakowitz , and R. Krull . 2013. “Comprehension of Viscous Morphology ‐ Evaluation of Fractal and Conventional Parameters for Rheological Characterization of *Aspergillus niger* Culture Broth.” Journal of Biotechnology 163, no. 2: 124–132. 10.1016/j.jbiotec.2012.08.027. 23059168

[bit28874-bib-0043] Yang, H. , U. Reichl , R. King , and E. D. Gilles . 1992. “Measurement and Simulation of the Morphological Development of Filamentous Microorganisms.” Biotechnology and Bioengineering 39, no. 1: 44–48. 10.1002/bit.260390108. 18600885

[bit28874-bib-0044] Zuccaro, A. , S. Götze , S. Kneip , P. Dersch , and J. Seibel . 2008. “Tailor‐Made Fructooligosaccharides by a Combination of Substrate and Genetic Engineering.” ChemBioChem 9, no. 1: 143–149. 10.1002/cbic.200700486. 18058889

