# Supplementary material for: Oxygen Consumption in Filamentous Pellets of Aspergillus niger: Microelectrode Measurements and Modeling
Source: Biotechnol Bioeng. 2024 Nov 4;122(2):306–21. doi: 10.1002/bit.28874 (PMC11718433; doi:10.1002/bit.28874)
Supplement: Supplementary file 1 — Supporting information. [file BIT-122-306-s001.docx]

**Supplementary Information to**

**Oxygen consumption in filamentous pellets of *Aspergillus niger*: Microelectrode measurements and modeling**

Charlotte Deffur**,** Anna Dinius, Julian Pagel, Henri Müller, Stefan Schmideder, Heiko Briesen and Rainer Krull

*Correspondence:*

Heiko Briesen ([heiko.briesen@tum.de](mailto:heiko.briesen@tum.de)), Chair of Process Systems Engineering, Technical University of Munich, Gregor-Mendel-Str. 4, 85354 Freising, Germany

Rainer Krull ([r.krull@tu-braunschweig.de](mailto:r.krull@tu-braunschweig.de)), Institute of Biochemical Engineering, Technische Universität Braunschweig, Rebenring 56, 38106 Braunschweig, Germany

**Table of Content**

1. Calculation of characteristic times for key processes
2. Pellet radii and total hyphal volume
3. Mean intensity projections of CT images
4. Measured oxygen concentration profiles and local hyphal fraction
5. Pellet Shape Analysis
6. Akaike information criterion
7. Individual estimated yield coefficients
8. Results of the Simulation of Oxygen Concentration
9. Reference

**1 Calculation of characteristic times for key processes**

In the following, the characteristic times for the processes relevant to this study were calculated to estimate the order of magnitude of how fast these processes occur. The key processes considered include oxygen consumption, diffusion into the pellet, and biological growth. Equation 1S calculates the time required for a culture to consume the entire saturation concentration of oxygen $c_{O_{2},saturation}$, assuming no oxygen is added, for example, by aeration, at a given oxygen uptake rate ($OUR$). Equation 2S provides an approximate estimate of the time required for a diffusive process to equilibrate a characteristic distance $R$ with concentration boundaries ranging from 0 to 100%. Equation 3S determines the time required for biomass to increase by a factor of $k$ at a given maximal growth rate $\mu_{max}$.

| $\tau_{OUR}=\frac{c_{O_{2},saturation}}{OUR}$ | (1S) |
| --- | --- |
| $\tau_{D_{O_{2},eff}}=\frac{R^{2}}{D_{O_{2},eff}}$ | (2S) |
| $\tau_{growth}=\frac{\ln(k)}{\mu_{max}}$ | (3S) |

Figure 1S compares the characteristic times for oxygen uptake, diffusion, and growth, as computed from Equations 1S, 2S, and 3S, for typical values, providing a comparative visualization under varying conditions.


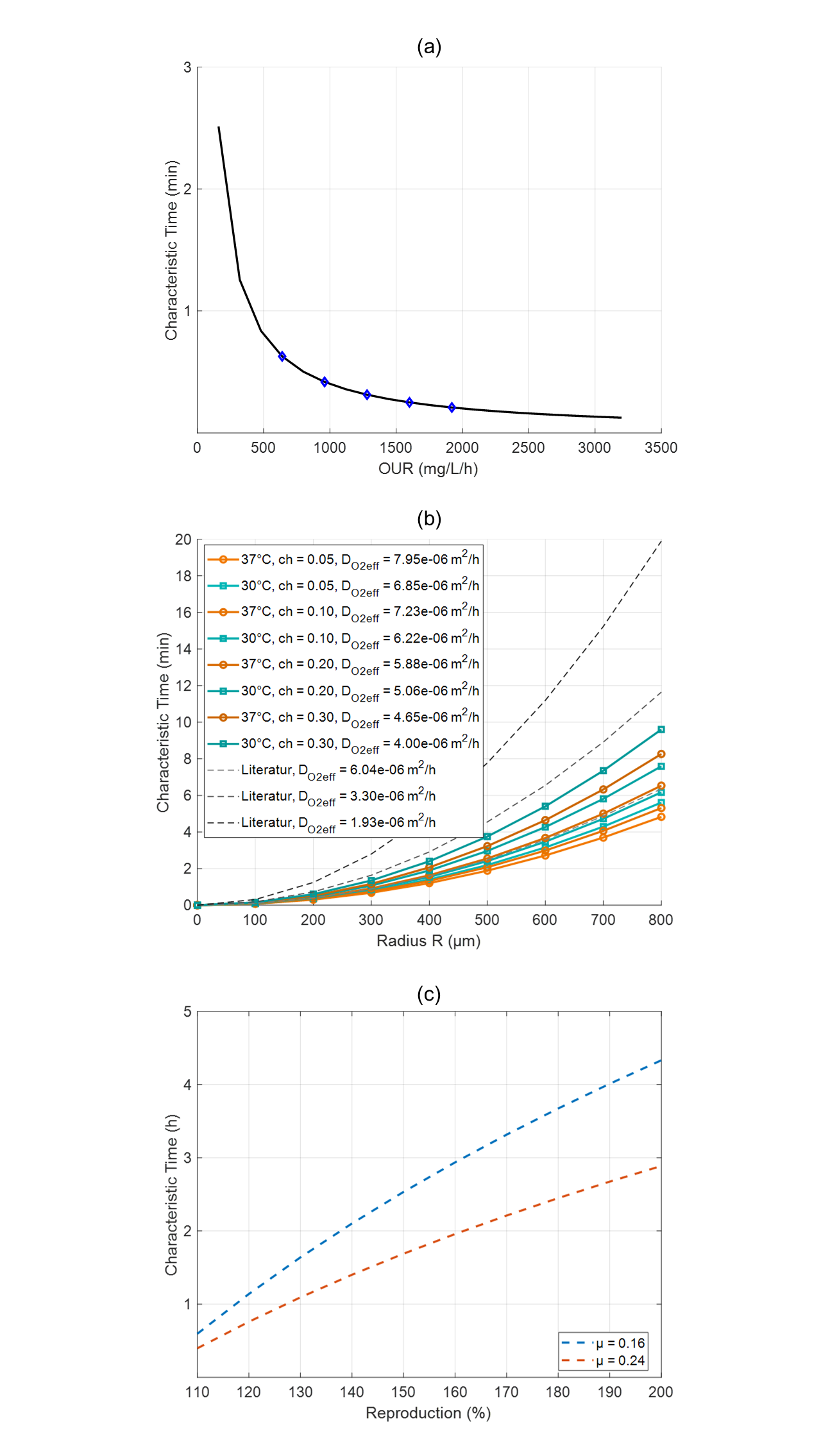


**Figure 1S:** Calculation of characteristic times. **(a**) $OUR$ values from Liu et al., 2017 were employed (marked in blue) and an oxygen saturation concentration of 6.7 mg/L was used for calculation. (b) The effective diffusion coefficient was calculated using the law from Equation 3 for realistic hyphal fractions (0.05 – 0.3) at both 37 and 30 °C. These values were compared with effective diffusion coefficients from the literature (Gonciarz, J., Bizukojc, M., 2014), which were determined at a temperature of 30 °C. (c) A $\mu_{max}$ of 0.16 $h^{-1}$ was used for strain R, and a $\mu_{max}$ of 0.24 $h^{-1}$ for strain H. A factor $k$ of 2, for example, corresponds to the doubling time, representing an increase to 200%.

**2 Pellet radii and total hyphal volume**

**Figure 2S**


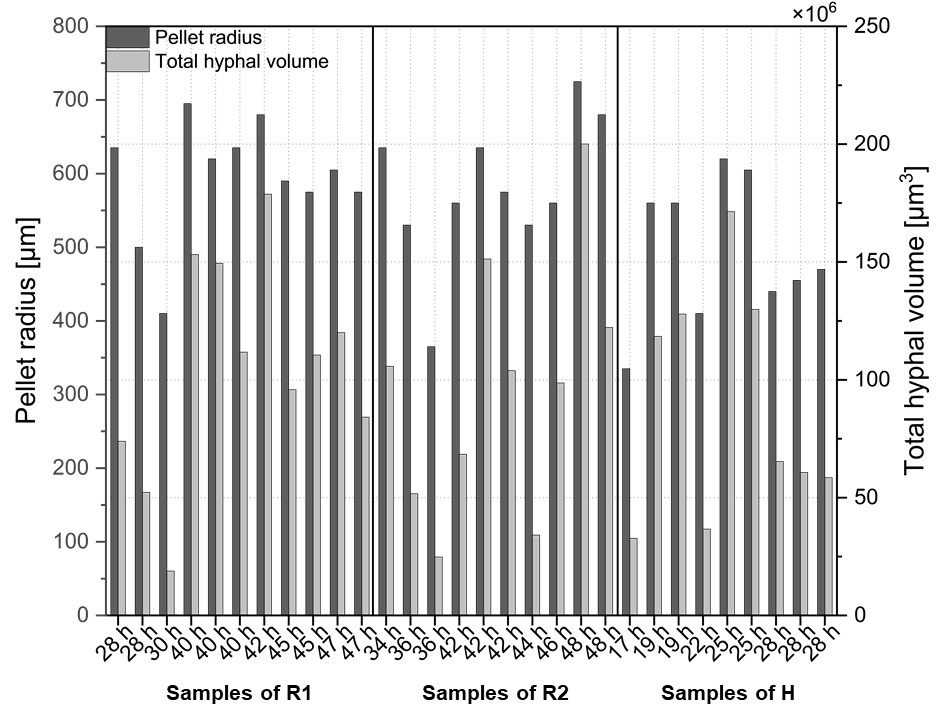


**Figure 2S** Overview of the individual pellet radii (dark grey) and total hyphal volume (light grey) for all examined pellets. Measurements were obtained using µCT and subsequent image processing.

**3 Mean intensity projections of CT images**

**Figure 3S**


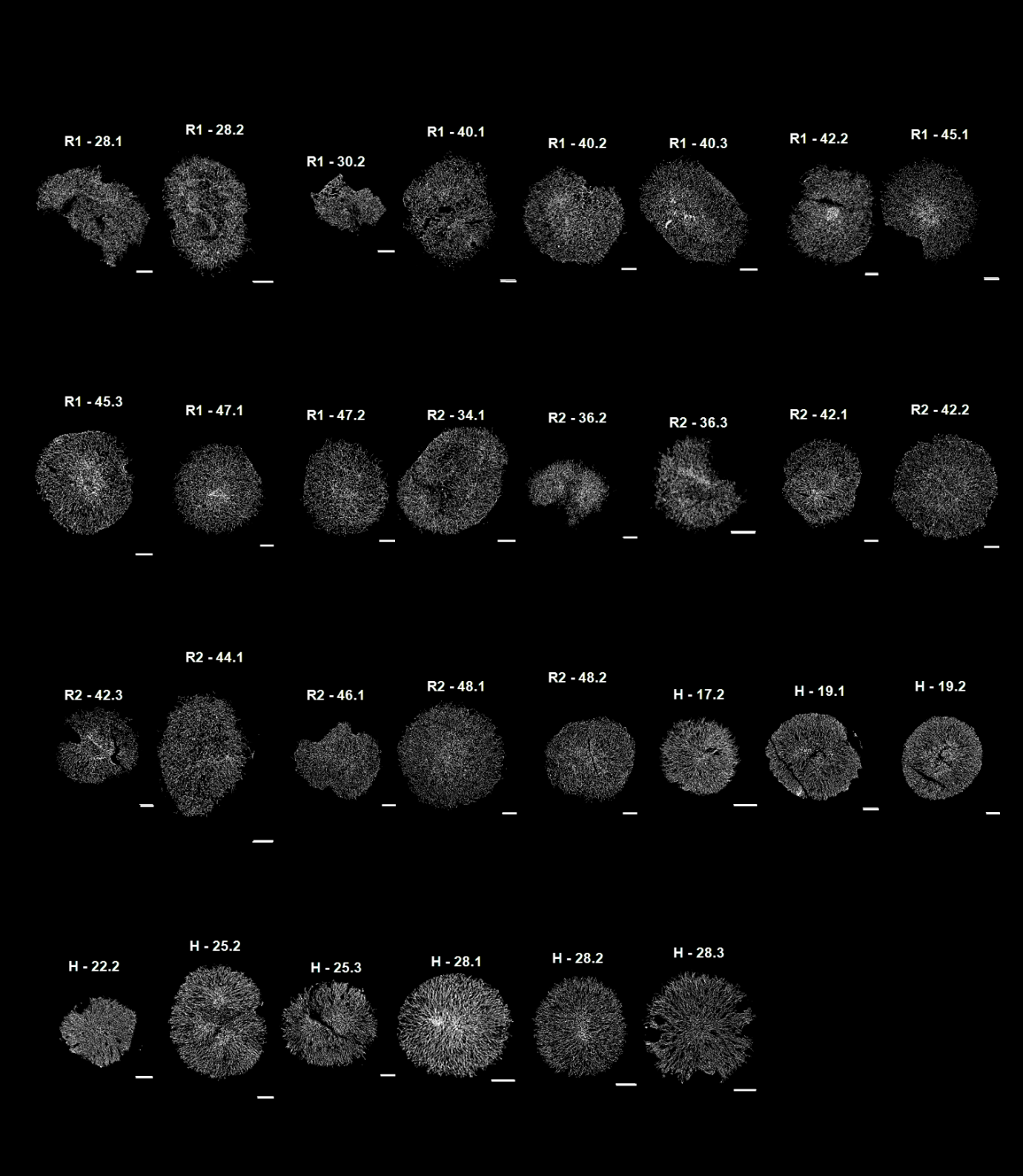


**Figure 3S:** Mean intensity projections of CT images for all pellets. Scale bar represents 250 µm. Sample name indicates cultivation code (R1, R2, H), sampling hour (18 - 48), and pellet number (1 - 3).

*For a higher resolution, please view the attached image.*

**4 Measured oxygen concentration profiles and local hyphal fraction**


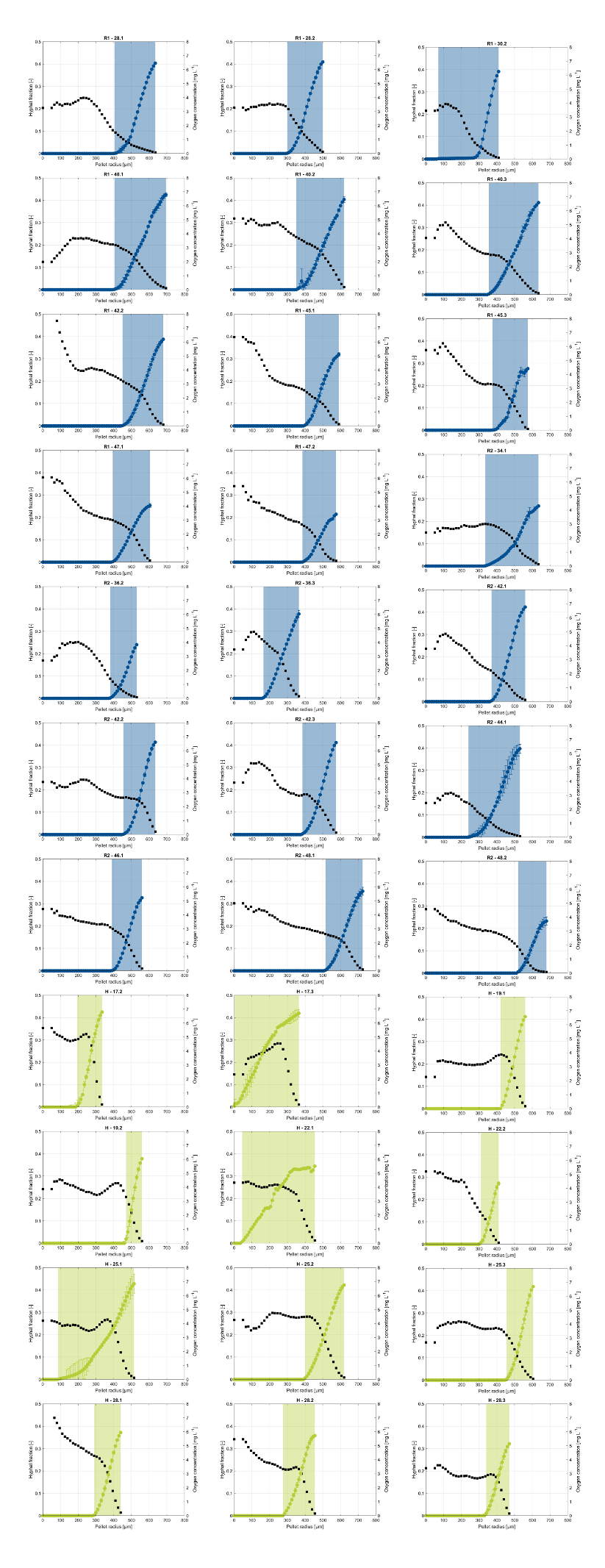


**Figure 4S:** Measured oxygen concentration profiles and local hyphal fraction in cultivation. For each pellet subjected to oxygen profiling, structural data analysis yields radius-resolved hyphal fractions (black plot), which are correlated with the measured oxygen concentration profiles (blue plot for strain R, green for strain H). The subplot title identifies individual pellets (e.g., “H - 28.2”: cultivation H, sample time 28 h, pellet number 2). Samples H 17.3, H 22.1, and H 25.1 were excluded from further analysis due to unreliable data, which is suspected to result from issues with the micromanipulator and the insertion process into the pellet. It is assumed that rather than being penetrated, the pellet was displaced.

*For a higher resolution, please view the attached image.*

**5 Pellet Shape Analysis**

**Tabelle 1S:** Classification of Pellets by sphericity and axis ratio of regular (R) and hyperbranching (H) strains. Sample name indicates cultivation code (R1, R2, H), sampling hour (18 - 48), and pellet number (1 -3).

| Sample name | Pellet sphericity | Axis ratio |
| --- | --- | --- |
| R1 - 28.1 | 0.91 | 0.50 |
| R1 - 28.2 | 0.93 | 0.55 |
| R1 - 30.2 | 0.86 | 0.39 |
| R1 - 40.1 | 0.96 | 0.77 |
| R1 - 40.2 | 0.96 | 0.74 |
| R1 - 40.3 | 0.96 | 0.73 |
| R1 - 42.2 | 0.97 | 0.82 |
| R1 - 45.1 | 0.96 | 0.89 |
| R1 - 45.3 | 0.97 | 0.78 |
| R1 - 47.1 | 0.96 | 0.83 |
| R1 - 47.2 | 0.95 | 0.73 |
| R2 - 34.1 | 0.93 | 0.56 |
| R2 - 36.2 | 0.93 | 0.52 |
| R2 - 36.3 | 0.94 | 0.72 |
| R2 - 42.1 | 0.90 | 0.58 |
| R2 - 42.2 | 0.97 | 0.90 |
| R2 - 42.3 | 0.98 | 0.33 |
| R2 - 44.1 | 0.87 | 0.39 |
| R2 - 46.1 | 0.96 | 0.84 |
| R2 - 48.1 | 0.98 | 0.94 |
| R2 - 48.2 | 0.95 | 0.68 |
| H - 17.2 | 0.97 | 0.89 |
| H - 19.1 | 0.98 | 0.79 |
| H - 19.2 | 0.98 | 0.75 |
| H - 22.2 | 0.90 | 0.52 |
| H - 25.2 | 0.98 | 0.75 |
| H - 25.3 | 0.97 | 0.78 |
| H - 28.1 | 0.97 | 0.73 |
| H - 28.2 | 0.98 | 0.90 |
| H - 28.3 | 0.99 | 0.93 |

**6 Akaike information criterion**

**Tabelle 2S:** Overview of model optimizations and Akaike Information Criterion results using Equation 5 (accounting for maintenance metabolism) or Equation 4 (consumption driven by biomass production). Each model employs distinct parameter estimation strategies to simulate oxygen profiles within fungal pellets. Default values refer to reference or literature-derived values initially utilized in the models. Optimized values are mean values obtained from parameter optimization for each model, indicating the refined estimates based on model fitting. The term "not optimized" is used to specify parameters that were kept at their default or literature values during the optimization process. The model with the smallest AICc (modified AIC for small sample sizes) is most favorable, which is marked in bold. RSS corresponds to the residual sum of squares.

|  |  | $Y_{X/{O_{2}}}$ | $m_{O_{2},max}$ | $c_{O_{2},crit}$ | $K_{XO}$ | $K_{MO}$ | $Y_{X/{O_{2}}}$ | $m_{O_{2},max}$ | $c_{O_{2},crit}$ | $K_{XO}$ | $K_{MO}$ | AIC | AIC_c_ | RSS |
| --- | --- | --- | --- | --- | --- | --- | --- | --- | --- | --- | --- | --- | --- | --- |
|  | Default values | $2.77$ | $7.2\times{10}^{-3}$ | $1\times{10}^{-5}$ | $2.5\times{10}^{-5}$ | $1.5\times{10}^{-5}$ |  |  |  |  |  |  |  |  |
|  |  | Optimized Values (mean values) | | | | | Spreading in all pellets (Standard deviation) | | | | |  |  |  |
| Model 1 | Equation 5 | $10.52$ | $0.10$ | $5.5\times{10}^{-4}$ | $3.9\times{10}^{-4}$ | $3.9\times{10}^{-5}$ | $7.49$ | $0.075$ | $7.3\times{10}^{-4}$ | $3.0\times{10}^{-4}$ | $3.4\times{10}^{-5}$ | $-254.46$ | $-247.46$ | $1.54\times{10}^{-5}$ |
| Model 2 | Equation 5 | $22.25$ | $0.11$ | $1.5\times{10}^{-3}$ | $7.5\times{10}^{-4}$ | not opti. | $4.85$ | $0.062$ | $7.6\times{10}^{-4}$ | $3.5\times{10}^{-4}$ | not opti. | $-258.40$ | $-253.78$ | $1.39\times{10}^{-5}$ |
| Model 3 | Equation 5 | $18.24$ | $0.10$ | $1.2\times{10}^{-3}$ | not opti. | not opti. | $7.92$ | $0.059$ | $9.6\times{10}^{-4}$ | not opti. | not opti. | $-260.34$ | $-257.48$ | $1.40\times{10}^{-5}$ |
| Model 4 | Equation 5 | $24.19$ | $0.11$ | not opti. | not opti. | not opti. | $0.85$ | $0.062$ | not opti. | not opti. | not opti. | $-262.74$ | $-261.14$ | $1.38\times{10}^{-5}$ |
| Model 5 | Equation 4 | 2.00 | - | - | $1.2\times{10}^{-5}$ | - | 0.72 | - | - | $1.6\times{10}^{-5}$ | - | $-$263.12 | $-$261.52 | $1.34\times{10}^{-5}$ |
| Model 6 | Equation 4 | 1.95 | - | - | not opti. | - | 0.72 | - | - | not opti. | - | $-$**263.82** | $-$**263.07** | $1.43\times{10}^{-5}$ |
| Model 7 | Equation 5 | not opti. | not opti. | $1.8\times{10}^{-5}$ | $4.9\times{10}^{-5}$ | $9.8\times{10}^{-5}$ | not opti. | not opti. | $1.4\times{10}^{-5}$ | $1.2\times{10}^{-4}$ | $2.5\times{10}^{-4}$ | $-216.63$ | $-$213.77 | $1.39\times{10}^{-4}$ |
| Model 8 | Equation 5 | not opti. | not opti. | not opti. | $6.2\times{10}^{-5}$ | $1.82\times{10}^{-4}$ | not opti. | not opti. | not opti. | $1.7\times{10}^{-4}$ | $3.1\times{10}^{-4}$ | $-218.44$ | $-216.84$ | $1.41\times{10}^{-4}$ |
| Model 9 | Equation 4 | not opti. | - | - | $3.3\times{10}^{-5}$ | - | not opti. | - | - | $1.0\times{10}^{-4}$ | not opti. | $-215.52$ | $-214.77$ | $1.82\times{10}^{-4}$ |

**7 Individual estimated yield coefficients**

**Figure 5S**
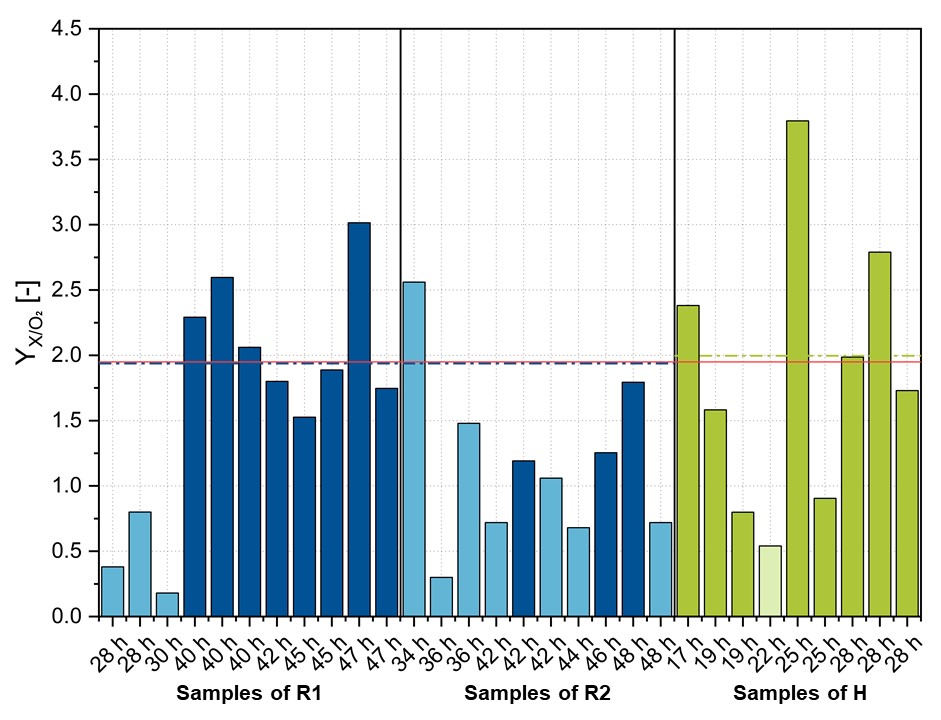


**Figure 5S** Yield coefficient $Y_{X/{O_{2}}}$ estimated individually for all examined pellets based on the experimental data of this study. Presented are the average $Y_{X/{O_{2}}}$ of all estimated pellets (red horizontal line), the average $Y_{X/{O_{2}}}$ of all pellets originating from cultivation R1 and R2 (dashed blue line) and the average $Y_{X/{O_{2}}}$ of all pellets originating from cultivation H (dashed green line).

**8 Results of the Simulation of Oxygen Concentration**


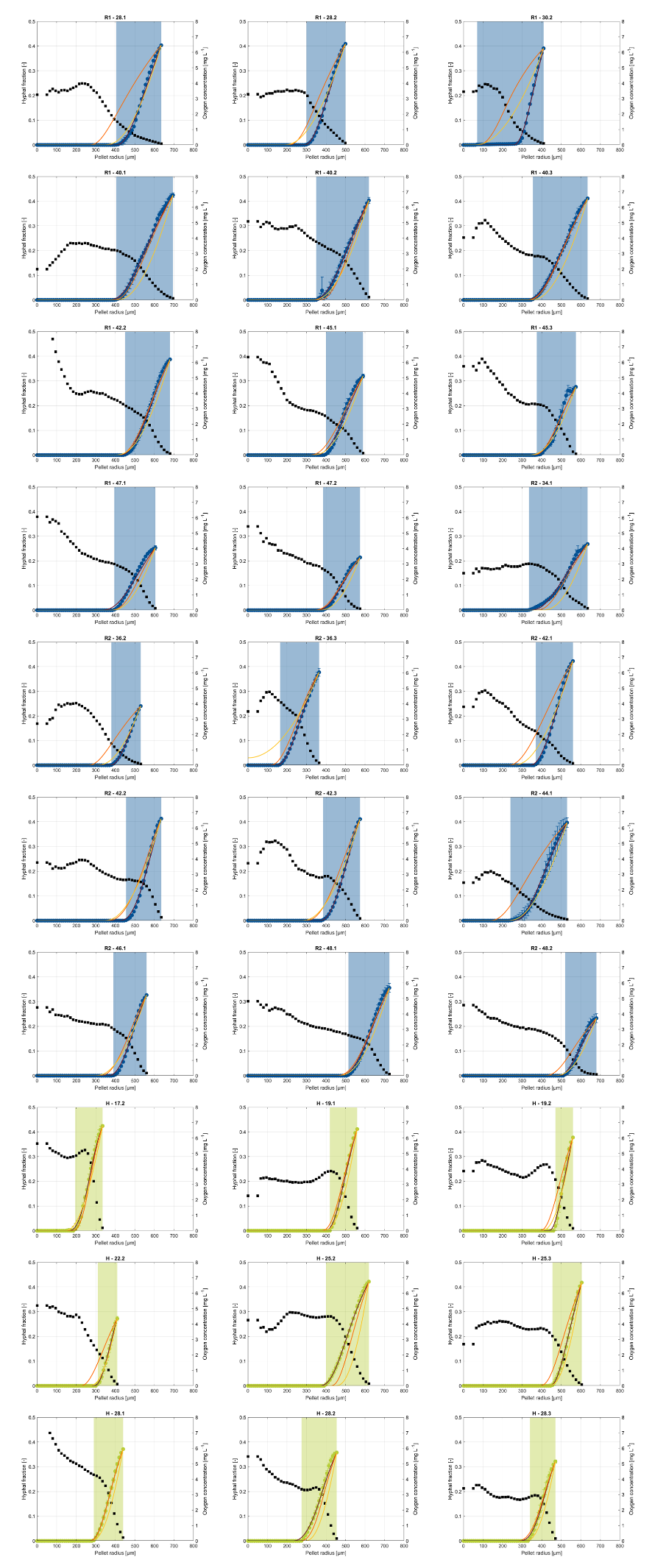


**Figure 6S:** Pellet data from 3D imaging, oxygen profiling, and simulations for strains R and H include radius-resolved hyphal fractions (black plot) correlated with oxygen concentrations (blue for R, green for H). Oxygen profiles were computed using: (1) using the pellet specific estimated parameter for calculating the oxygen concentration in individual pellets (red); (2) using all pellets´ mean value for calculating the oxygen concentration in individual pellets (orange); (3) using a constant hyphal fraction in the pellet, averaged over all pellets within each strain for calculating the oxygen concentration in individual pellets (yellow). The subplot title identifies individual pellets (e.g., “H - 28.2”: cultivation H, sample time 28 h, pellet number 2).

*For a higher resolution, please view the attached image.*

**References**

Gonciarz, J., & Bizukojc, M. (2014). Adding talc microparticles to *Aspergillus terreus* ATCC 20542 preculture decreases fungal pellet size and improves lovastatin production. Engineering in Life Sciences, 14(2), 190–200. https://doi.org/10.1002/elsc.201300055

Liu, X., Tian, X., Hang, H., Zhao, W., Wang, Y., & Chu, J. (2017). Influence of initial glucose concentration on seed culture of sodium gluconate production by *Aspergillus niger*. Bioresources and Bioprocessing, 4(1). DOI:10.1186/s40643-017-0185-1
